# Probabilistic fluorescence-based synapse detection

**DOI:** 10.1371/journal.pcbi.1005493

**Published:** 2017-04-17

**Authors:** Anish K. Simhal, Cecilia Aguerrebere, Forrest Collman, Joshua T. Vogelstein, Kristina D. Micheva, Richard J. Weinberg, Stephen J. Smith, Guillermo Sapiro

**Affiliations:** 1 Electrical and Computer Engineering, Duke University, Durham, North Carolina, United States of America; 2 Synapse Biology, Allen Institute for Brain Sciences, Seattle, Washington, United States of America; 3 Department of Biomedical Engineering, Johns Hopkins University, Baltimore, Maryland, United States of America; 4 Molecular and Cellular Physiology, Stanford University School of Medicine, Stanford, California, United States of America; 5 Department of Cell Biology and Physiology, University of North Carolina, Chapel Hill, Chapel Hill, North Carolina, United States of America; 6 Department of Biomedical Engineering, Department of Computer Science, Department of Mathematics, Duke University, Durham, North Carolina, United States of America; Northeastern University, UNITED STATES

## Abstract

Deeper exploration of the brain’s vast synaptic networks will require new tools for high-throughput structural and molecular profiling of the diverse populations of synapses that compose those networks. Fluorescence microscopy (FM) and electron microscopy (EM) offer complementary advantages and disadvantages for single-synapse analysis. FM combines exquisite molecular discrimination capacities with high speed and low cost, but rigorous discrimination between synaptic and non-synaptic fluorescence signals is challenging. In contrast, EM remains the gold standard for reliable identification of a synapse, but offers only limited molecular discrimination and is slow and costly. To develop and test single-synapse image analysis methods, we have used datasets from conjugate array tomography (cAT), which provides voxel-conjugate FM and EM (annotated) images of the same individual synapses. We report a novel unsupervised probabilistic method for detection of synapses from multiplex FM (muxFM) image data, and evaluate this method both by comparison to EM gold standard annotated data and by examining its capacity to reproduce known important features of cortical synapse distributions. The proposed probabilistic model-based synapse detector accepts molecular-morphological synapse models as user queries, and delivers a volumetric map of the probability that each voxel represents part of a synapse. Taking human annotation of cAT EM data as ground truth, we show that our algorithm detects synapses from muxFM data alone as successfully as human annotators seeing only the muxFM data, and accurately reproduces known architectural features of cortical synapse distributions. This approach opens the door to data-driven discovery of new synapse types and their density. We suggest that our probabilistic synapse detector will also be useful for analysis of standard confocal and super-resolution FM images, where EM cross-validation is not practical.

## Author summary

Brain function results from communication between neurons connected by complex synaptic networks. Synapses are themselves highly complex and diverse signaling machines, containing protein products of hundreds of different genes, some in hundreds of copies, precisely arranged at each individual synapse. Synapses are fundamental not only to synaptic network function but also to network development, adaptation, and memory. In addition, abnormalities of synapse numbers or their molecular components have been implicated in a variety of mental and neurological disorders. Despite their obvious importance, mammalian synapse populations have so far resisted detailed quantitative study. In human brains and most animal nervous systems, synapses are very small and very densely packed: there are approximately 1 billion synapses per cubic millimeter of human cortex. This volumetric density poses very substantial challenges to proteometric analysis at the critical level of the individual synapse. The present work describes new probabilistic image analysis methods suitable for single-synapse analysis of synapse populations in both animal and human brains, in health and disorder.

## Introduction

Deeper understanding of the basic mechanisms and pathologies of the brain’s synaptic networks will require advances in our quantitative understanding of structural, molecular, and functional diversity within the vast populations of individual synapses that define those networks [1] [2] [3] [4]. Regardless of the subject of interest, synapse heterogeneity makes assay at the single-synapse level paramount. Here, we introduce and characterize a novel image analysis method for automated detection and molecular measurement of individual synapses and single-synapse molecular profiling of diverse synapse populations from multiplex fluorescence microscopy (muxFM) image data. The proposed methodology for structural identification and molecular analysis of single synapses at scale will be an enabling step toward deeper experimental analysis of the relationships between synaptic structure, molecules, and function. Reliable, high-throughput methods for large-scale synapse detection will also help to analyze volume images large enough to contain complete neural arbors, and thus to allow discernment of the relationships between detected synapses and their presynaptic and postsynaptic parent neurons [5].

The synapse detection methodology described here is not the first to grapple with the challenges of detecting synapses in immunofluorescence images [6] [7] [8] [9] [10]. The special utility and novelty of this tool partially lies in (1) producing outputs in the form of probability maps, reflecting the limited certainty with which synapses can be detected by most experimental modalities [11], and (2) the superior utility for both interactive and algorithmic exploration which is conferred by the query-based architecture resulting from the **unsupervised** framework. The probabilistic detection algorithm we introduce has perhaps its closest precedent in probabilistic synapse detectors that were introduced recently for the analysis of Focussed Ion Beam Scanning Electron Microscope (FIBSEM) images [12] [13]. The relationship in particular with [8] will be further discussed later in this paper.

Single-synapse profiling of large and diverse synapse populations poses formidable challenges [14] [15] [16]. Electron microscopy (EM) of appropriately labeled specimens defines the current ‘gold standard’ for synapse detection: the nanometer resolution of EM is necessary for the unambiguous identification of defining synaptic features such as presynaptic vesicles, synaptic clefts, and postsynaptic densities [17] [18]. Unfortunately EM data acquisition is technically difficult, slow, burdened by large data processing and storage requirements, and offers only limited capacities to discriminate amongst the hundreds of different synaptic proteins that constitute the synaptic proteome. In contrast, fluorescence microscopy (FM) of tagged specimens is much faster and less expensive, easier to segment for analysis, and offers much greater molecular discrimination power. Unfortunately, the ability of FM to detect and discriminate individual synapses is compromised by resolution limits and the close crowding of synapses in most neural tissue specimens of interest. Robust FM detection of synapses is nonetheless potentially possible by combining measures that extend resolution limits and multiplexing for localization and co-localization of synaptic markers.

In designing the algorithm and software reported here, we first relied on images acquired with conjugate array tomography (cAT), which combines the strengths of FM-AT with those of electron microscopic array tomography (EM-AT), allowing both EM and muxFM imaging of individual cortical specimens. Array tomography’s ultrathin physical sectioning provides z-axis resolution far beyond the light microscopic diffraction limit, as well as high sensitivity and high lateral resolution, while greatly simplifying voxel-conjugate registration of FM-AT and EM-AT images. FM-AT can moreover multiplex large numbers of synaptic markers by its combination of sequential and spectral label multiplexing. Thus, cAT provides an ideal platform for the development and rigorous design and testing of algorithms aimed at single-synapse molecular analysis and population molecular profiling.

The remarkable structural and molecular diversity within mammalian synapse populations challenges our present biological understanding of how to define a synapse [11]. Difficulties also arise from a very broad distribution of synapse size, with the smallest synapses occurring at the highest frequencies. Thus, detection of a synapse inevitably involves setting some minimum-size criterion for any candidate cell-cell contact specialization to qualify as a synapse. For FM, the ‘size’ metric is typically the intensity of one or more fluorescent synaptic protein tags. The fact that there are clearly non-synaptic ‘backgrounds’, and that the observed size distributions are log-normal, enforces high sensitivity of synapse detection on some rather arbitrary threshold minimum size value. This sensitivity in turn makes key results of widespread interest, such as the synaptic density in a region or the presence/absence of a synapse at a given microscopic site, uncomfortably dependent on that same size threshold value. The probabilistic synapse detector proposed here may lead both to relief from such arbitrary-threshold (parameters) and to improvements in our biological understanding of what defines a synapse.

The unsupervised probabilistic synapse detector reported here accepts molecular-morphological synapse models in the form of user queries, and delivers a volumetric map of the probability that each voxel represents part of a synapse. These maps can then be used directly to detect, classify, and map putative synapses, with confidence statistics for each. Taking human annotation of cAT EM data as ground truth, we show that our algorithm detects synapses from muxFM data alone as effectively as human annotators (while seeing only the muxFM data), and can reproduce known architectural features of cortical synapse distributions. The algorithm is actually validated with the most comprehensive AT datasets currently available. Though we here address only array tomography image data, our probabilistic synapse detector may also be useful for analysis of widely available confocal and super-resolution FM images.

## Methods

### Overview

**Fig 1:**
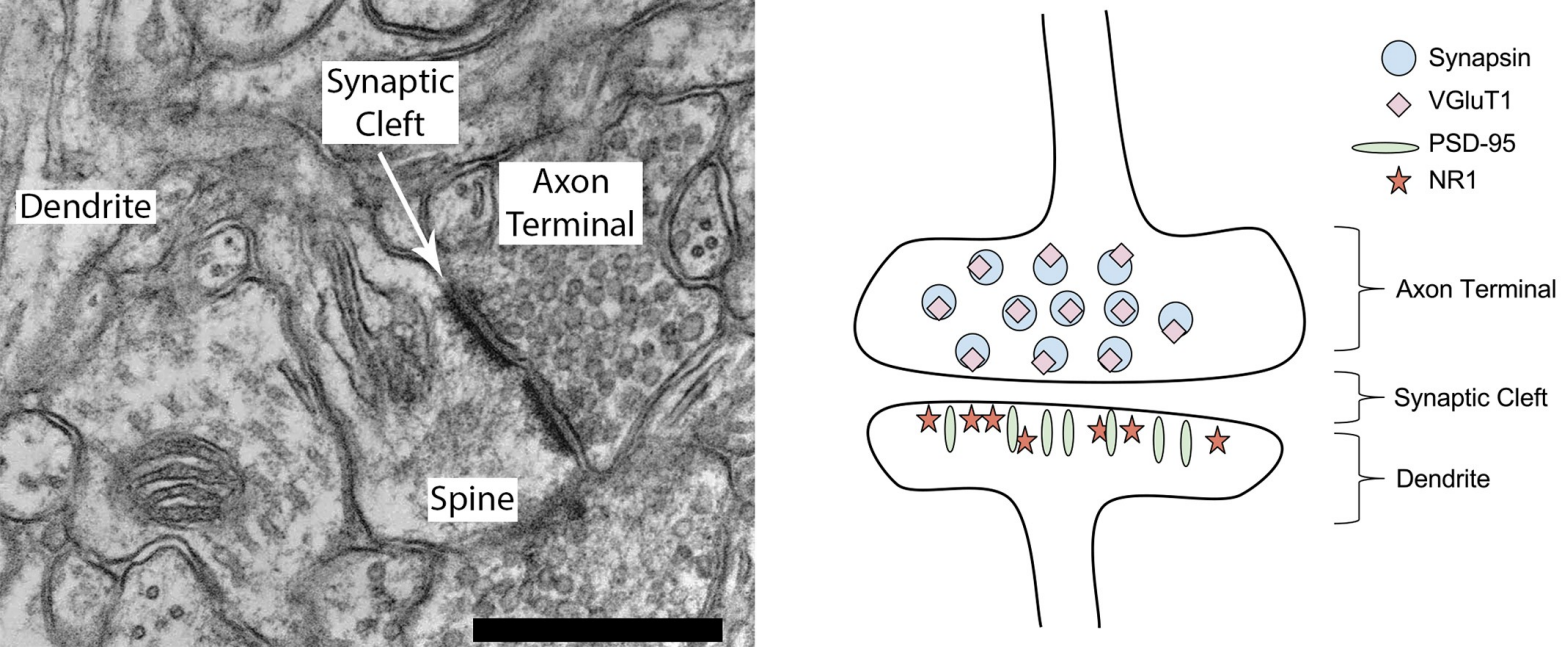
Excitatory synapse. **Left**: A typical axospinous synapse (in the lower right-hand region of the electron micrograph). An axon terminal packed with small round vesicles of neurotransmitter (right) is closely apposed to a dendritic spine; at the junction a slightly increased electron density on the presynaptic plasma membrane (‘presynaptic active zone’) is precisely matched across the about 30 nm wide synaptic cleft by a dark extension into the dendritic spine, the ‘postsynaptic density.’ This synapse is perforated (the slight break in increased density halfway along the synapse). The membranous structure within the spine head is a ‘spine apparatus.’ Because of a fortunate plane of section, the plasma membrane of this spine is continuous with its parent dendritic shaft (left edge of photo), which contains longitudinally-sectioned microtubules. The scale bar represents 500 nm. **Right**: Cartoon diagramming the molecular architecture of an excitatory PSD-95-expressing synapse [19]. Basic biological knowledge about synapse structure and protein composition as depicted in this figure is used to inform the proposed query-based probabilistic algorithm.

**Fig 2:**
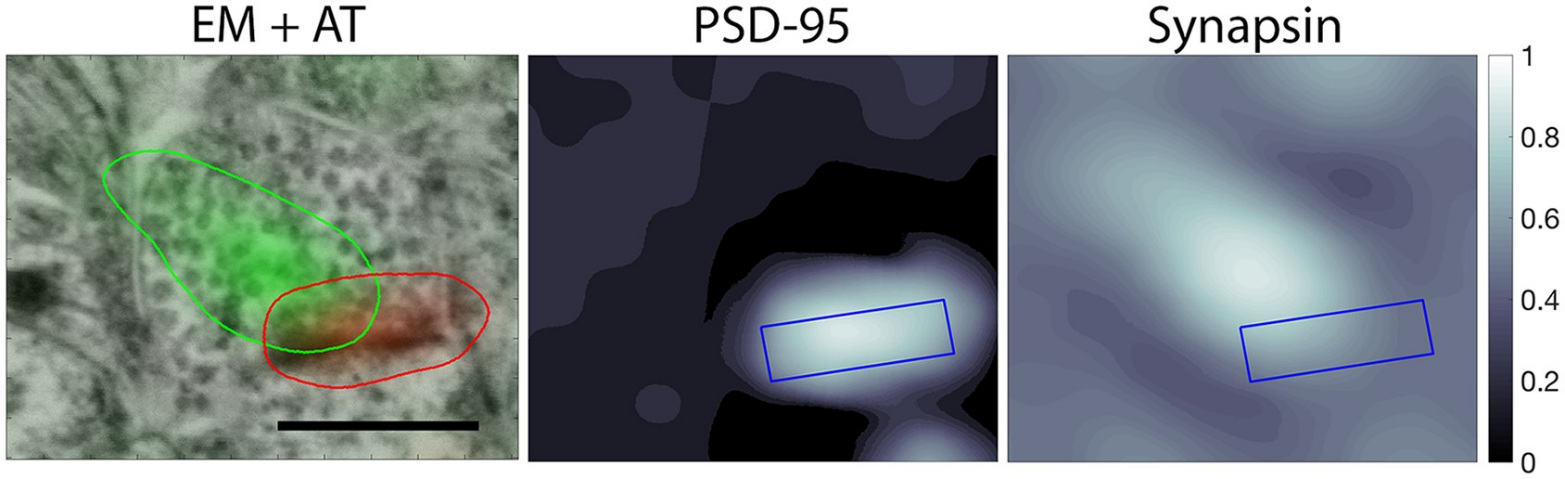
cAT data. Three 1.277 *μ* m ×1.186 *μ* m images showing conjugate EM and IF data. The left panel has synapsin (green) and PSD-95 (red) data overlaid, marked by the colored boundary lines. The scale bar represents 500 nm. The presence of both presynaptic and postsynaptic channels indicates the presence of a synapse with high probability. The center panel shows the PSD-95 IF image and the right panel shows the synapsin IF image. On both images, the EM-identified synaptic cleft is marked by a blue box. While the proposed synapse detection method uses multiple synaptic markers, only two are shown here for visualization purposes.

The proposed algorithm is inspired from biological knowledge of synapse characteristics. Synapses include two major structural components: a presynaptic terminal and a postsynaptic terminal. Detecting synapses using data from immunofluorescence imaging involves identifying such adjacent presynaptic and postsynaptic antibody markers, as shown in Fig 1, which diagrams the locations of four major excitatory synaptic proteins. Fig 2 is an example of an excitatory synapse with images of presynaptic and postsynaptic antibody markers (synapsin and PSD-95) overlaid upon an EM image. For this example, only two antibody markers are shown for visual simplicity—in practice, any number of presynaptic or postsynaptic antibody markers may be used by the proposed algorithm for synapse detection.

Manual synapse identification involves determining the punctum size and brightness in one channel, and then considering adjacency to similarly-defined puncta in other channels. However, without corresponding EM data, detections using only IF data have an associated degree of uncertainty. Thus, we propose a query-based probabilistic synapse detection method that reflects the thought process underlying expert manual synapse detection.

The first step is to distinguish signal from background noise. This calculation encodes the probability that the pixel value represents authentic antigen detection. The second step is to determine whether the foreground pixels correspond to a 2D punctum, since photons emanating from only a single pixel usually reflect noise. Therefore, adjacent ‘positive’ pixels, more likely to reflect a synaptic punctum, are augmented. Third, puncta that span multiple slices have a higher probability of belonging to a synapse than those that do not. To visualize this effect, the probability of a punctum belonging to a synapse is attenuated based on whether the prospective punctum spans multiple slices. The last step in computing the synapse probability map is to evaluate the presence of adjacent presynaptic and postsynaptic puncta by correlating the corresponding IF volumes. This produces a probability map, where the value at each voxel is the probability it belongs to a synapse. This algorithm provides a general framework for the evaluation of a wide variety of synapse subtypes, user-defined by setting the presynaptic and postsynaptic antibodies and puncta size.

**Fig 3:**
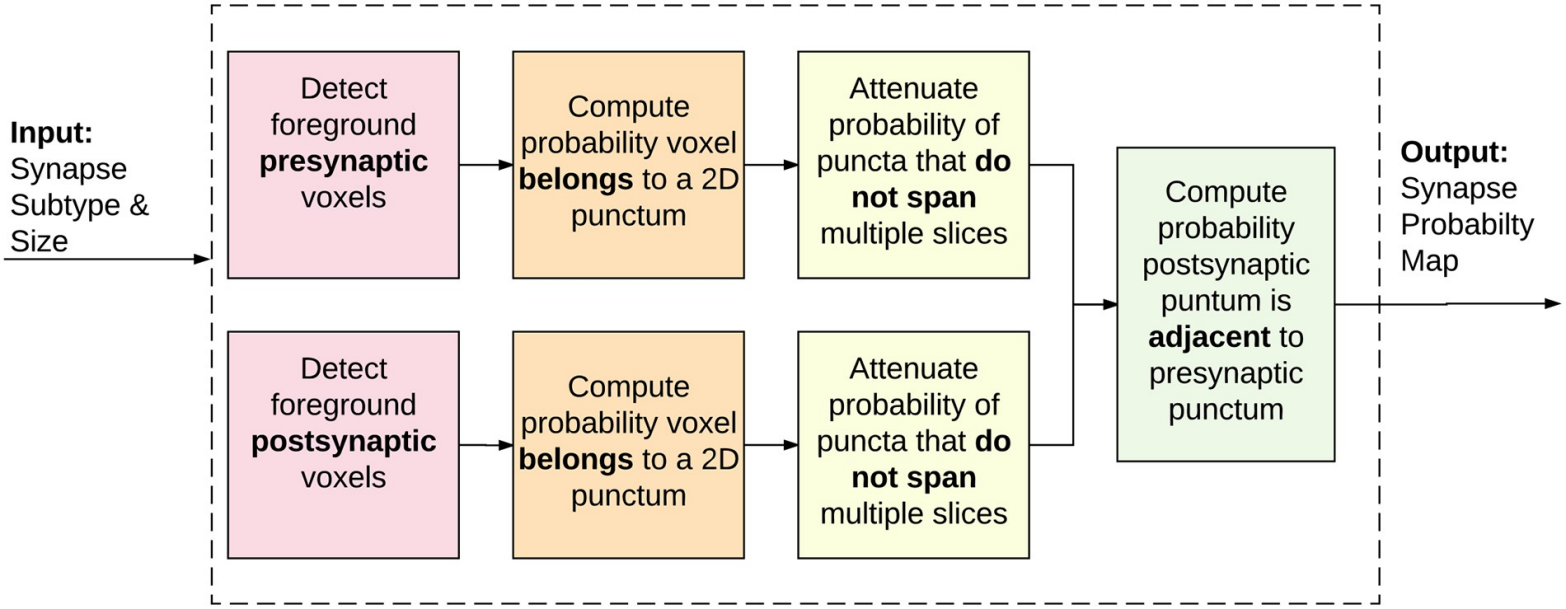
Proposed method flowchart. Fundamental steps of the proposed probabilistic synapse detection algorithm.

The following sections describe in detail each step in the process, as diagrammed in Fig 3. Before that, let us relate our proposed approach with the current state-of-the-art method [8], which inspired this work. The method in [8] requires manually annotating a large number of excitatory synapses using the EM data, and then using this as labeled data for training (**supervised training**). EM data allows the user to differentiate between symmetric and asymmetric synapses, but does not allow for subtype identification (limited labels/supervision). Thus, the support vector machine (SVM) classifier used in [8] is trained with synapses containing the marker for PSD-95, but does not take into account synapses without the PSD-95 marker (limited classes in the supervision). In contrast, the approach here proposed and detailed below is **unsupervised**, allowing the user to detect synapses with multiple proteometric compositions without first using other methods to identify large numbers of synapses for training. Our proposed approach does not require the user to manually inspect associated EM for training data; in fact, we do not require associated EM data with the IF data. Instead, we enable the user to ‘define’ (biologically inspired) a synapse by specifying which synaptic markers should be present (query) and what the minimum size of those markers should be, allowing a more class specific synaptic search. This is critical also for the discovery of new types of synapses, exploitation of new markers, and data-based discovery. The method in [8] will need re-training for every new class they want to find in the IF (potentially even new data acquisition protocols as well). There is a direct numerical comparison of the two methods in the experimental section, showing that the proposed algorithm is not only unsupervised and more broadly applicable than [8], but it actually outperforms it in the cases where both methods can be used.

### Step 1: Computation of foreground probability

Raw immunofluorescence image data is noisy; for example, speckles of the antibody markers often bind with cellular structures not associated with synapses, such as mitochondria. In addition, fluorescence imagery contains signal from sources other than fluorescently-labelled antibodies, e.g. from background autofluorescence. Finally, all digital imagery contains inherent noise from sources such as camera read noise and photon shot noise. The noise produced by these sources is usually smaller in magnitude than that originating from authentic synaptic labeling, but it cannot simply be filtered out and dismissed from consideration, since the signal may originate from a true synaptic site, and we want to allow for the possibility that a concordance of weak evidence will lead to the detection of a synapse. Thus, the first step of the algorithm consists of differentiating the bright voxels, the foreground (potential objects of interest), from a noisy background in a probabilistic fashion.

**Fig 4:**
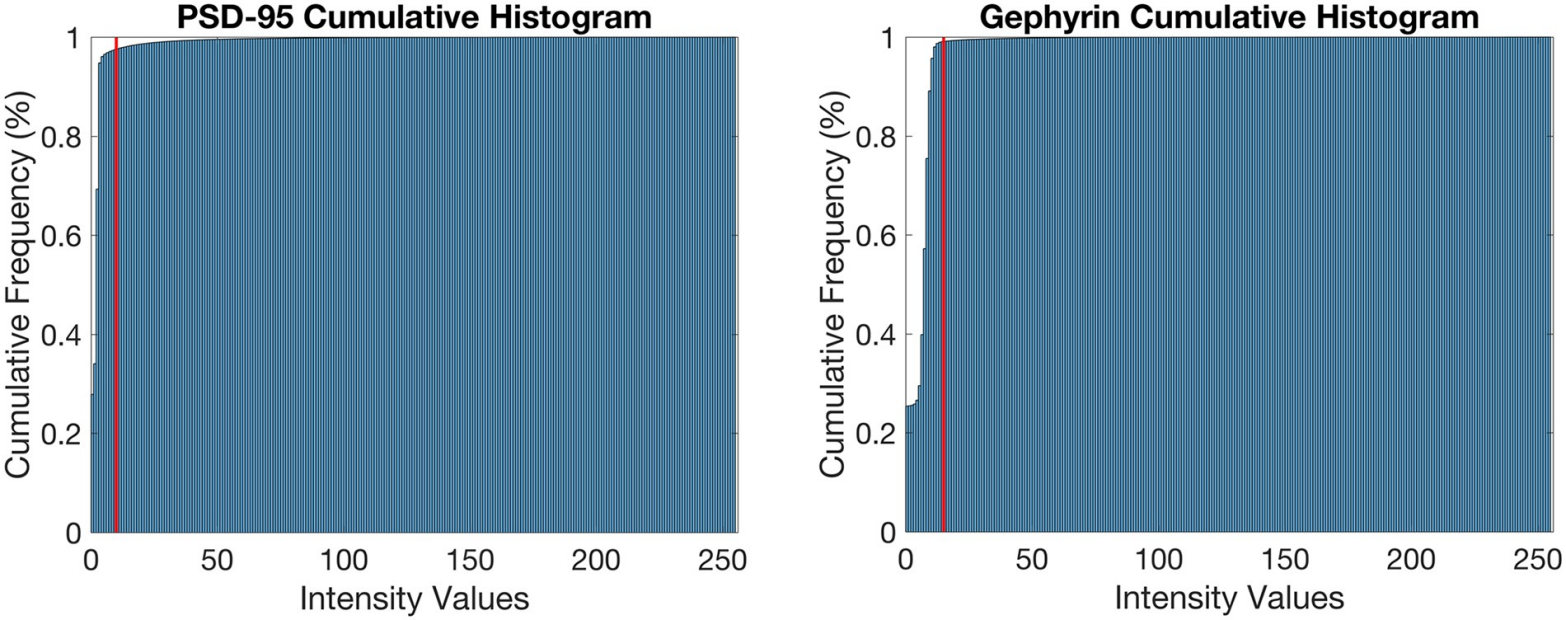
Raw IF data histograms. These cumulative histograms show that typically ∼98% of voxels in the dataset lie below the threshold line indicated in red. The threshold lines are estimates based on visual inspection of the data.

IF data volumes, when stained for synaptic markers, are also extremely sparse—approximately 2% of the voxels in the dataset belong to the foreground, as indicated in Fig 4. Therefore, the IF image volume can be used to approximate the distribution of the background noise.

Let *v*(*x*, *y*, *z*) be the intensity value of a voxel at position (*x*, *y*) in slice *z*, for a given channel of the IF data. A probabilistic model, *p*_*B*_, is computed which characterizes all the pixels that belong to the background, which includes approximately 98% of the voxels. The background noise model is computed independently for each slice to account for variations in tissue and imaging properties. The background model *p*_*B*_ is assumed to be a Gaussian distribution, whose mean and variance $\left(\mu_{B},\sigma_{B}^{2}\right)$ are empirically computed from each slice *z* (the *z* index is omitted in Eq 1 for simplicity of notation). Then, the probability of a voxel belonging to the background, i.e. not being ‘bright’, is given by

$$\begin{array}{c} p_{B}\left(x,y,z\right)=\frac{1}{\sigma_{B}\sqrt{2\pi }}\int_{v(x,y,z)}^{\infty }e^{\frac{-{\left(t-\mu_{B}\right)}^{2}}{2\sigma_{B}^{2}}}dt. \end{array}$$ (1)

Therefore, the probability of a voxel associated with the foreground, *p*_*F*_, is computed as

$$\begin{array}{c} p_{F}\left(x,y,z\right)=1-p_{B}\left(x,y,z\right). \end{array}$$ (2)

**Fig 5:**
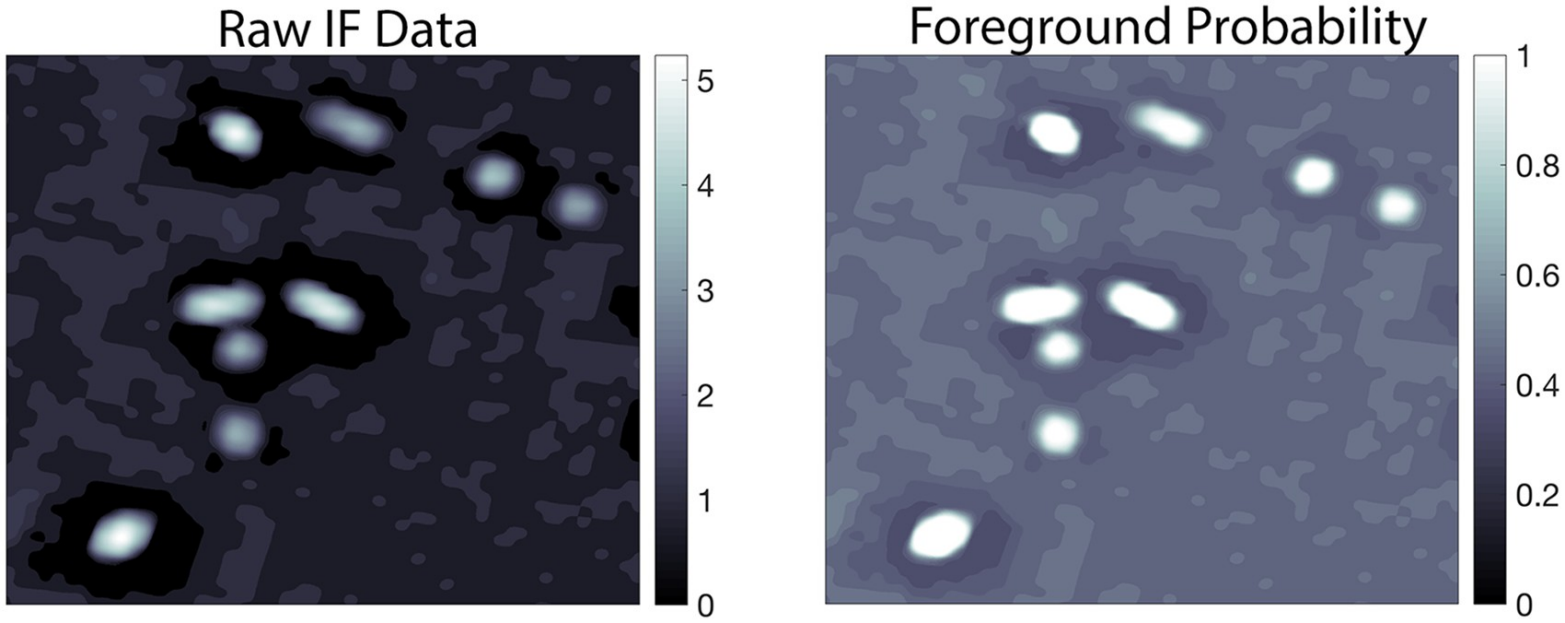
Foreground probability. Cutout of size 2261×2501 pixels or 5.268×5.827*μ* m of the logarithm (for visualization purposes) of the IF raw data (left) and the corresponding image of the foreground probability map (right) of one slice of the PSD-95 antibody. The proposed approach clearly differentiates the high probability bright pixels from the background low probability pixels. The ‘dark’ rings around the puncta are an artifact of the deconvolution performed prior to image alignment, and its spatial extent has been taken into account in the spatially-oriented next steps of the algorithm. The AT data appears ‘quantized’ because it has been upsampled from its native 100 nm per pixel resolution to 2.33 nm per pixel to align the AT data with the EM data.

Fig 5 shows an example of the transformation from the raw data to the foreground probability map.

### Step 2: Probability of 2D puncta

Once foreground pixels have been identified in a probabilistic fashion, the next step is to determine if they form a 2D punctum. Since synapses appear as bright puncta in the IF image data, voxels which form puncta should have a higher probability of being associated with a synapse than those which do not. The probability of a voxel belonging to a 2D punctum, *p*_*P*_, is computed by multiplying the voxel’s foreground probability by that of its neighbors in a predefined neighborhood region,

$$\begin{array}{c} p_{P}\left(x,y,z\right)=\prod_{i=x-W}^{x+W}\prod_{j=y-W}^{y+W}p_{F}\left(i,j,z\right), \end{array}$$ (3)

where *W* is the neighborhood size, defined by the smallest expected punctum size. These operations are analogous to applying a box filter on the logarithm of the probability map, for computational efficiency. In our experiments, *W* was set to be slightly larger than the size of the point spread function of the microscopes used.

**Fig 6:**
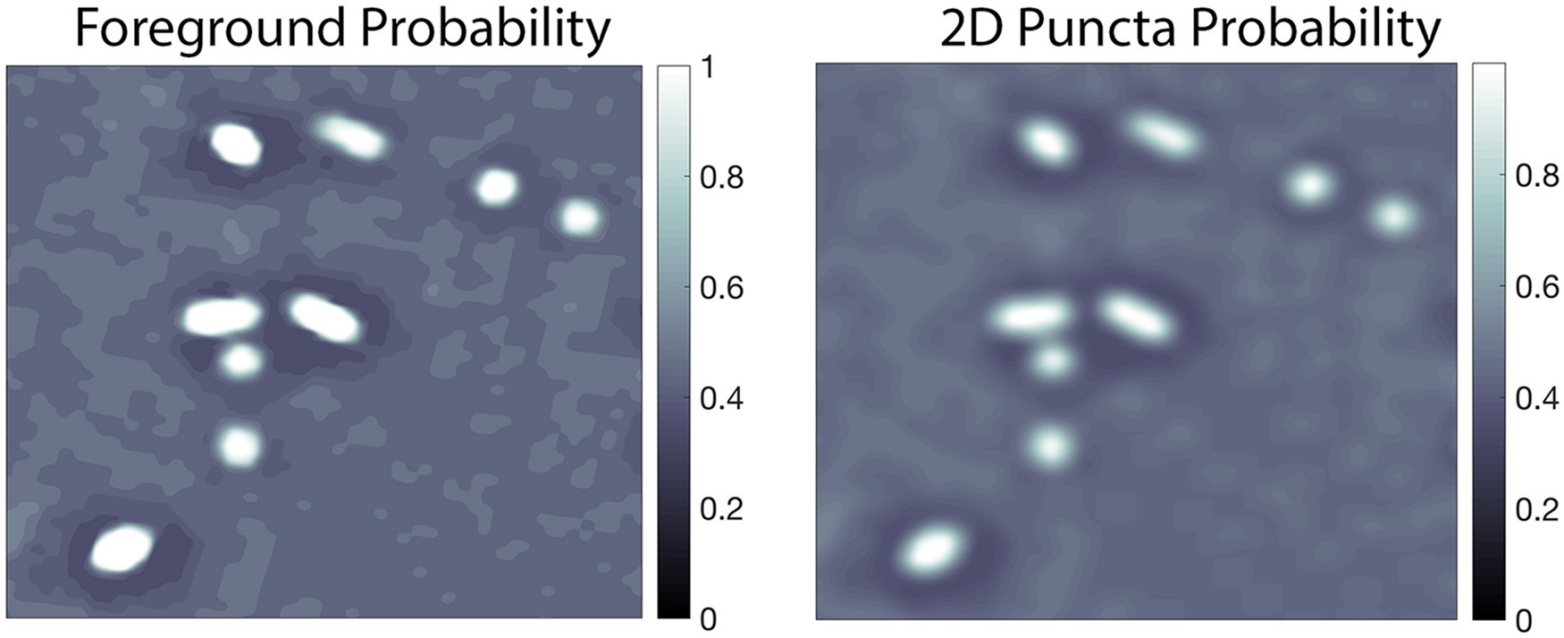
2D puncta probability. The image on the left is the output from step one, a portion of a single slice of PSD-95 where each pixel codes the probability that it represents signal, not noise. The image on the right is the result of the corresponding probability map of each pixel belonging to a 2D punctum. Both images are cutouts of size 2261 × 2501 pixels or 5.268 × 5.827*μ* m.

Fig 6 shows an example of the foreground probability map and the corresponding 2D puncta probability map. This step transforms each pixel’s value from representing the probability it belongs to the foreground to the probability it belongs to both the foreground and to a punctum.

### Step 3: Probability of 3D puncta

Potential synaptic puncta can span multiple slices of a given channel; puncta that span multiple slices have a higher probability of being associated with a synapse than those that do not. Therefore, we propose a factor *f*(*x*, *y*, *z*) which diminishes the probability values associated with voxels which do not maintain a similar probability value in adjacent slices,

$$\begin{array}{c} f\left(x,y,z\right)=\exp\left\{-\sum_{j=j_{start}}^{j=j_{end}}{\left[p_{P}\left(x,y,z\right)-p_{P}\left(x,y,z+j\right)\right]}^{2}\right\}. \end{array}$$ (4)

**Fig 7:**
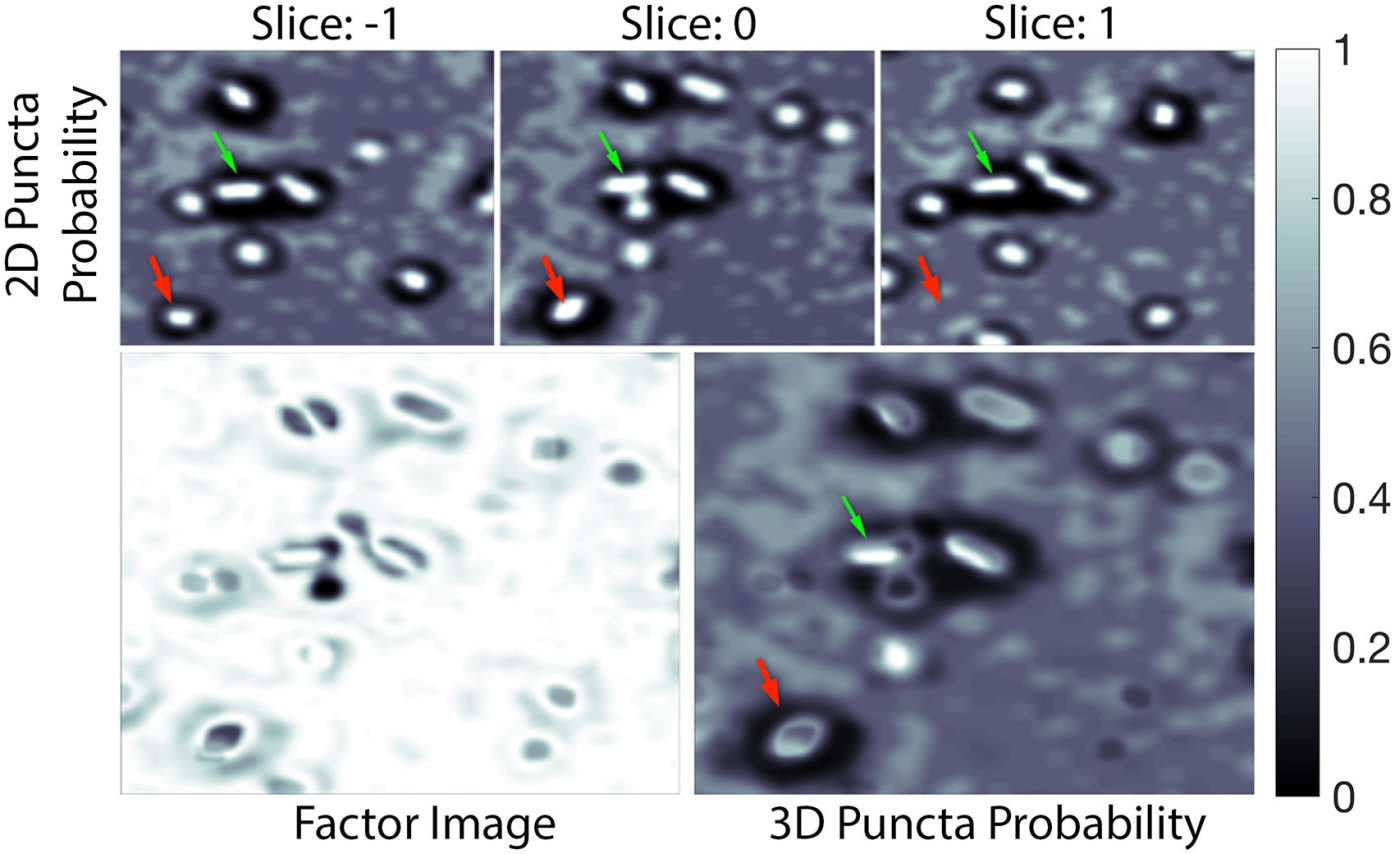
3D puncta probability. **Top:** Three consecutive slices of 2D puncta probability, given by Eq (3). **Bottom:** Factor image given by Eq (4) (left) and the corresponding 3D puncta probability, given by Eq (5), (right) of the center slice in the top row. Only those 2D puncta that actually span multiple slices are kept with high intensity (probability) in the combined result (bottom right). The green arrow points to an example of a probable punctum that spans multiple slices. The red arrow points to an example of a relatively-less probable punctum which does not span multiple slices and therefore is diminished in the output image. Each image is a cutout of size 2261 × 2501 pixels or 5.268 × 5.827*μ* m.

The pixel’s 2D puncta probability is compared to that of its neighbor in slice(s) before, *j*_*start*_, and slice(s) after, *j*_*end*_. The number of slices compared is dependent on the input size parameter for each antibody. The factor attenuates values for 2D puncta that do not span the required number of slices, as shown in Fig 7.

The 3D puncta probability map is then computed by multiplying the 2D puncta probability map by this factor,

$$\begin{array}{c} p_{3DP}\left(x,y,z\right)=p_{P}\left(x,y,z\right)f\left(x,y,z\right), \end{array}$$ (5)

which further improves the probability of a detection by considering the slice-to-slice spatial distribution, going from 2D to 3D.

### Step 4: Adjacency of presynaptic and postsynaptic puncta

**Fig 8:**
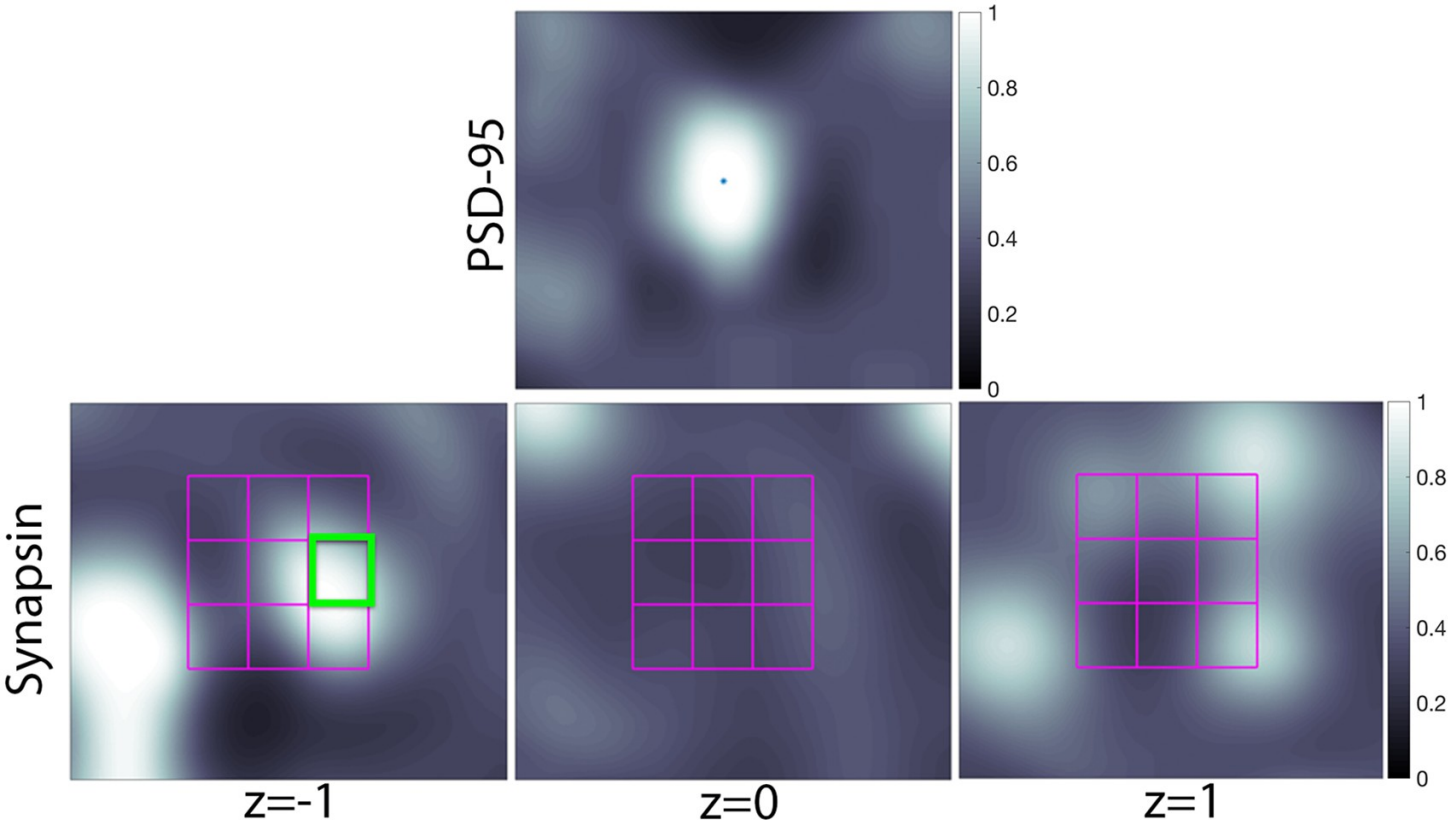
Presynaptic and postsynaptic puncta adjacency. The first row contains a cutout showing a PSD-95 punctum with a pixel highlighted in the center of the image. The second row contains synapsin cutouts with the search grid overlaid. For this example, *K* = 3, so shown is a 3 × 3 grid spanning 3 slices is shown. The brightest box is highlighted in green. Thus, the output value of the synaptic probability map at the pixel specific in the PSD-95 image is the average pixel value of the green box multiplied by the intensity value of the PSD-95 pixel.

In electron microscopic images, synapses are identified by the presence of synaptic vesicles on the presynaptic side, the close adjacency of the membranes of the presynaptic axon terminal to a postsynaptic dendrite or dendritic spine, and the presence of a distinct postsynaptic specialization, as diagrammed in Fig 1. Synapses are identified in IF data by the close spatial arrangement of pre- and postsynaptic antibody markers, which correspond to proteins associated with synapses. Therefore, the next step in our approach is to look for the presence of presynaptic puncta in the neighborhood of postsynaptic puncta. More precisely, for each postsynaptic antibody voxel (i.e., PSD-95), we search in the adjacent 3D neighborhood of the corresponding presynaptic (i.e., synapsin) volume for a high intensity probability signal. To accomplish this, a rectangular grid is defined in the presynaptic channels around each postsynaptic voxel, as shown in Fig 8. The size of the grid is defined by the initial query parameters, which depend on both the inherent biology and microscope resolution. The logarithm of the 3D puncta probability map Eq (5) is integrated in each grid location and the maximum is taken as presynaptic signal level around the given postsynaptic location,

$$\begin{array}{c} \log p_{pres}=\max_{k}\left(\sum_{G_{k}}\log p_{3DP}\left(presynaptic\right)\right), \end{array}$$ (6)

where the grid *G* is centered at the current voxel (*x*, *y*, *z*) and divided into *K* × *K* × *K* subregions *G*_*k*_. To search in a grid around a defined voxel location for the presynaptic signal, *K* is set to 3. When searching for the postsynaptic signal, *K* is set to 1 since postsynaptic signals are expected to loosely co-localize. These values can be adopted to the data resolution. The postsynaptic antibody pixel probability

$$\begin{array}{c} p_{post}=p_{3DP}\left(postsynaptic\right), \end{array}$$ (7)

is multiplied by the presynaptic probability to obtain the desired probability map:

$$\begin{array}{c} p_{synap}\left(x,y,z\right)=p_{pres}\left(x,y,z\right)p_{post}\left(x,y,z\right). \end{array}$$ (8)

**Fig 9:**
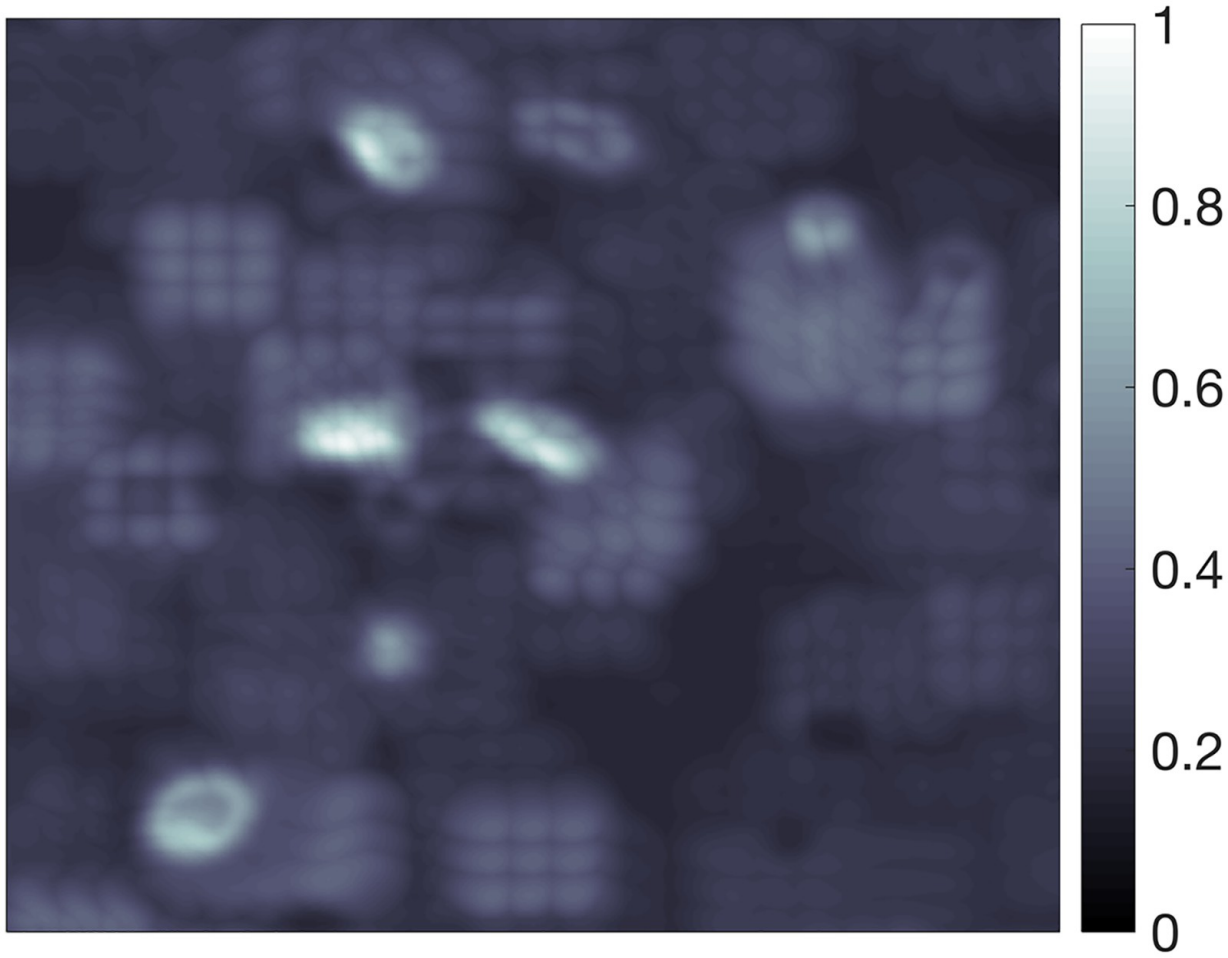
Synapse probability map. The output of the method, as described in Eq 8, where value at each voxel is the probability it belongs to a specific synapse subtype. Cutout of size 2261 × 2501 pixels or 5.268 × 5.827*μ* m.

Again, the probability information is here maintained (Fig 9), now including the morphological relationship between the channels. This ‘grid’ like approach allows the method to be robust to slight image alignment and registration issues, as well as to deconvolution artifacts.

## Results

The proposed method was evaluated on a series of array tomography (AT) datasets published in [8] and [19]. These datasets were acquired using the AT methods described in [20]. Each dataset was stained and imaged with antibodies for presynaptic and postsynaptic proteins and then aligned and registered. In the conjugate AT (cAT) dataset, the tissue samples were also imaged with a scanning electron microscope (SEM) and then the IF data were up-sampled, aligned, and registered to the EM data [8]. Synapses identifiable in the EM image data were labeled and used to provide ground truth. Table 1 lists the synaptic markers used. Synapsin is ubiquitous in both excitatory and inhibitory synapses; therefore, it is used as a presynaptic marker for excitatory and inhibitory queries. PSD-95, the postsynaptic density marker used here, is generically considered a reliable marker of excitatory synapses [8], [21], [20]. For each tissue sample, there were multiple antibody staining cycles and each cycle contained up to three different antibodies. Each round of staining included the fluorescent DNA stain DAPI, which helped facilitate the registration and alignment process. The exact overview of the sequence of antibody application can be found in [8] and [19].

**Table 1:** Synaptic markers used in this work across the various datasets. Not all markers were present in each dataset. Details, including the order of antibody application, can be found in [8] and [19].

Synapses		Antigen	Host	Antibody Source	RRID
All	Presynaptic	Synapsin 1	Rabbit	Cell Signaling Technology 5297	RRID:AB_2616578
Excitatory	Presynaptic	VGluT1	Guinea pig	Millipore AB5905	RRID:AB_2301751
		VGluT2	Guinea pig	Millipore AB2251	RRID:AB_1587626
	Postsynaptic	PSD-95	Rabbit	Cell Signaling Technology 3450	RRID:AB_2292883
		NR1	Mouse	Millipore MAB363	RRID:AB_94946
		NR2B	Mouse	NeuroMab 75-101	RRID:AB_2232584
		GluA1	Rabbit	Millipore AB1504	RRID:AB_2113602
		GluA2	Mouse	NeuroMab 75-002	RRID:AB_2232661
Inhibitory	Presynaptic	GABA	Guinea pig	Millipore AB175	RRID:AB_91011
		vGAT	Mouse	Synaptic Systems 131-011	RRID:AB_887872
		GAD	Rabbit	Cell Signaling Technology 5843	RRID:AB_10835855
	Postsynaptic	Gephyrin	Mouse	BD Biosciences 612632	RRID:AB_399669
		GABAARa1	Mouse	NeuroMab 75-136	RRID:AB_2108811

All the primary antibodies used are from commercial sources (see Table 1) and have been thoroughly characterized in previous work. The authors in [8] and [19] performed AT-specific controls described in detail in [20] [19]. Such controls include, but are not limited to, comparison with a different antibody for the same or similar antigen to test for the specificity of staining, comparison between adjacent sections to test the consistency of staining, and comparison with an antibody against a spatially exclusive antigen or nuclear label to evaluate background staining. Highly cross-adsorbed secondary antibodies of the appropriate species were used, such as ThermoFisher Scientific A-11029, A-11032, and A-21236 for detecting mouse primary antibodies. The application of these antibodies without a primary antibody did not result in any labeling.

### Evaluation on conjugate array tomography

#### Experimental setup

The proposed method was first evaluated on the cAT dataset published in [8] using the associated EM image data to create the ‘ground truth’ needed for evaluation. The datasets themselves are described in Table 2. To evaluate the method’s performance on excitatory synapses, the set of query parameters in Table 3 were used. For inhibitory synapse detection, the queries listed in Table 4 were used. These parameters were based on prior literature concerning synaptic proteins and their respective antibodies [7] [19]. Only 20 inhibitory synapses were manually identified in the KDM-SYN-120905 dataset; therefore, inhibitory synapse detection performance is only reported for the larger KDM-SYN-140115 dataset. For evaluation and visualization purposes, the output probability map, *p*_*synap*_(*x*, *y*, *z*), from each query was thresholded, and adjacent voxels that lie over the threshold were grouped into detections.

**Table 2:** The cAT datasets used for analysis [8].

	Dimension (Pixels)	Resolution	Labeled Synapses
**KDM-SYN-120905**	4508 × 6306 × 27	2.33 × 2.33 × 70 nm/pixels	236
**KDM-SYN-140115**	7936 × 9888 × 39	3.72 × 3.72 × 70 nm/pixels	1457

**Table 3:** Excitatory synapse detection queries for the cAT data. Note that the size dimension in *x*, *y* correspond to the window width *W* in Eq (3) and the *z* range corresponds to the number of slices, *j*, mentioned in Eq (4).

Query	Presynaptic		Postsynaptic	
	Antibody	Puncta Size (x,y,z) *μm*	Antibody	Puncta Size (x,y,z) *μ* m
1	synapsin	0.2 x 0.2 x 0.21	PSD-95	0.2 x 0.2 x 0.21
2	synapsin VGluT1	0.2 x 0.2 x 0.21 0.2 x 0.2 x 0.21	PSD-95	0.2 x 0.2 x 0.07
3	synapsin	0.2 x 0.2 x 0.21	PSD-95 NR1	0.2 x 0.2 x 0.07 0.2 x 0.2 x 0.07
4	synapsin VGluT1	0.2 x 0.2 x 0.21 0.2 x 0.2 x 0.21	PSD-95 NR1	0.2 x 0.2 x 0.07 0.2 x 0.2 x 0.07
5	synapsin VGluT1	0.2 x 0.2 x 0.21 0.2 x 0.2 x 0.07	PSD-95	0.2 x 0.2 x 0.07
6	synapsin VGluT1	0.2 x 0.2 x 0.07 0.2 x 0.2 x 0.21	PSD-95	0.2 x 0.2 x 0.07
7	synapsin	0.2 x 0.2 x 0.21	PSD-95 NR1	0.2 x 0.2 x 0.07 0.2 x 0.2 x 0.21

**Table 4:** Inhibitory synapse detection queries for the cAT data.

Query	Presynaptic		Postsynaptic	
	Antibody	Puncta Size (x,y,z) *μm*	Antibody	Puncta Size (x,y,z) *μ* m
1	synapsin GABA	0.2 x 0.2 x 0.21 0.2 x 0.2 x 0.07	gephyrin	0.2 x 0.2 x 0.07
2	synapsin VGAT	0.2 x 0.2 x 0.21 0.2 x 0.2 x 0.07	gephyrin	0.2 x 0.2 x 0.07
3	synapsin GAD	0.2 x 0.2 x 0.21 0.2 x 0.2 x 0.07	gephyrin	0.2 x 0.2 x 0.07

#### Performance metrics

The ground truth used in this work is obtained from the EM data since it represents the current ‘gold standard’ for manual synapse identification. Prior to imaging with scanning electron microscope (SEM), the tissue was embedded in Lowicryl, which preserves fine ultrastucture detail [22]. Not every synapse present in the tissue is identifiable with EM data, and not every synapse is marked with the antibodies used (that is, not identifiable with IF data, the only input for our algorithm). Consequently, there are synapses whose presence may be inferred with the IF data, but cannot be validated by visual inspection of the EM data. Similarly, there are synapses which are visually identifiable in the EM data, but, for a variety of reasons, were not stained by the antibody markers. These are examples of data points for which validation with images only (EM or IF) is not possible, and there is no expectation of an IF-based algorithm to detect/reject. These edge cases, which were excluded from evaluation, were estimated to be less than 10% of the total population of synapses.

We report in Table 5 the precision and recall values obtained for these two tested datasets. We differentiate two cases: first, considering all synapses manually identified in the EM data and counting all false positives returned by the program (referred to in Table 5 as ‘EM’); and second, considering the subset of detections that can be manually verified by an expert using only IF data (referred to in Table 5 as ‘IF’). For example, detections that the EM data lists as a false positives but are impossible to verify using only IF data are removed from evaluation. Similarly, manually-identified synapses in the EM data which do not appear in IF data are also removed from secondary evaluation.

**Table 5:** Results of excitatory and inhibitory synapse detection. Precision is defined as the number of true positives detections / (true positive detections + false positive detections). Recall is defined as the number of true synapses detected / (true synapses detected + missed synapses). The value after the precision-recall values is the 95% confidence interval as computed by the Agresti-Coull method [23].

**Dataset**	**Excitatory**			
	**EM**		**IF**	
	**Precision**	**Recall**	**Precision**	**Recall**
KDM-SYN-120905	0.88 ± 0.037	0.91±0.053	0.90±0.035	0.93±0.049
KDM-SYN-140115	0.92±0.016	0.94±0.017	0.93±0.015	0.95±0.016
**Dataset**	**Inhibitory**			
	**EM**		**IF**	
	**Precision**	**Recall**	**Precision**	**Recall**
KDM-SYN-120905	-	-	-	-
KDM-SYN-140115	0.82±0.062	0.81±0.088	0.91±0.049	0.91±0.072

#### Results

**Fig 10:**
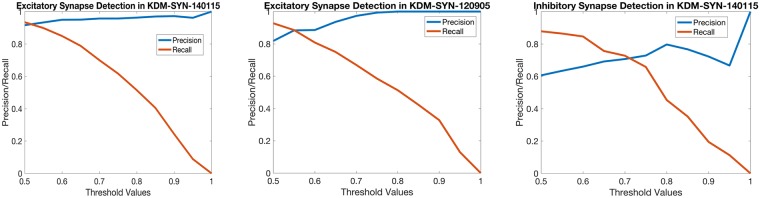
Precision/Recall curves. The relationship between the precision and recall values across a series of thresholds for each cAT dataset. As detailed in the text, the threshold is for validation purposes, since the proposed framework outputs a confidence/probability.

Once the final probability map for each query was computed, maps for excitatory synapses were thresholded at 0.6 for the KDM-SYN-140115 dataset and 0.55 for the KDM-SYN-120905 dataset. The maps for inhibitory synapses were thresholded at 0.7. These thresholds were based on the intersection of the precision/recall curves in Fig 10. The difference among threshold values likely reflects the different signal/noise distributions of each antibody. Note that this threshold, the only non-biological parameter of the system, can be ignored when working directly on the output (Eq 8), or easily set for the entire dataset by visually inspecting a few detections. Fig 10 shows the relationship between the final threshold and accuracy in greater detail. As shown in Table 5, the proposed algorithm successfully detects most synapses in both datasets, with only a small fraction of false positive detections. Based on the *IF only* indicator, we observe that the algorithm performs at human level (approximately 90% accuracy), with false positives and false negatives limited to cases which human experts (including co-authors of this manuscript) are also not confident of their own result [8].

As shown in Table 6, the proposed algorithm performs as well as the state-of-the-art method for excitatory synapse detection [8], while eliminating the need to undergo the labor-intensive process of cultivating a training dataset. Furthermore, due to the approximate ten-to-one ratio of excitatory to inhibitory synapses, creating training sets for inhibitory synapses is difficult. Our method is insensitive to the number of synapses per class as it only returns possible synapses which match the query parameters. Finally, the fact that we can skip training also makes the proposed system more applicable to diverse datasets without the need for re-design the entire process, as here demonstrated.

**Table 6:** State-of-the-art detection results for excitatory synapses from [8]. The value after the precision-recall values is the 95% confidence interval as computed by the Agresti-Coull method [23].

Excitatory, EM		
Dataset	Precision	Recall
KDM-SYN-120905	0.96±0.014	0.88±0.059
KDM-SYN-140115	0.94±0.0096	0.82±0.025

**Fig 11:**
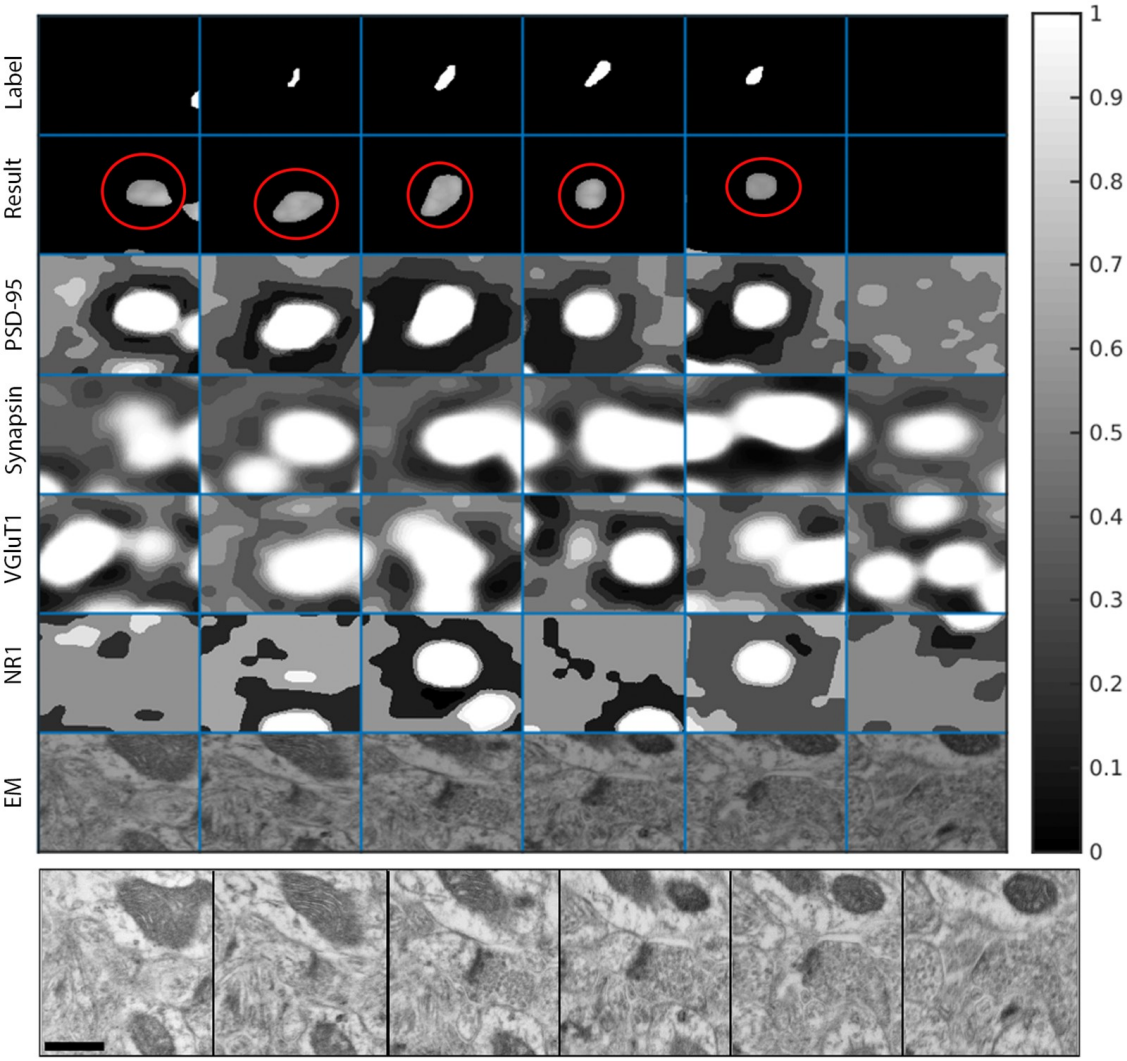
True positive synaptogram. Synaptogram showing the distribution of IF data for an EM identified synapse. Each column represents a single slice of data in the *z* direction, for a total of six slices. The first row (from the top) shows a manually labeled synaptic cleft, as identified in the EM volume. The EM data was used only for validation, since the method operates solely on the IF data. The second row shows the thresholded output of the proposed method, circled in red. Rows 3-6 show the corresponding foreground probability maps for each channel. PSD-95 is Postsynaptic Density 95, VGluT1 is Vesicular Glutamate Transporter 1, and NR1 is N-methyl-D-aspartate Receptor 1. PSD-95 and NR1 are both postsynaptic markers and tend to co-localize, while synapsin and VGluT1 are both presynaptic markers and tend to co-localize. The last row in the first panel shows the corresponding EM data. Each ‘block’ is 1.221*μm* × 1.233*μm*. The bottom panel shows enlarged, consecutive slices of the EM data, which was used to manually annotate the synapse. The scale bar on the lower left side is 500 nm.

**Fig 12:**
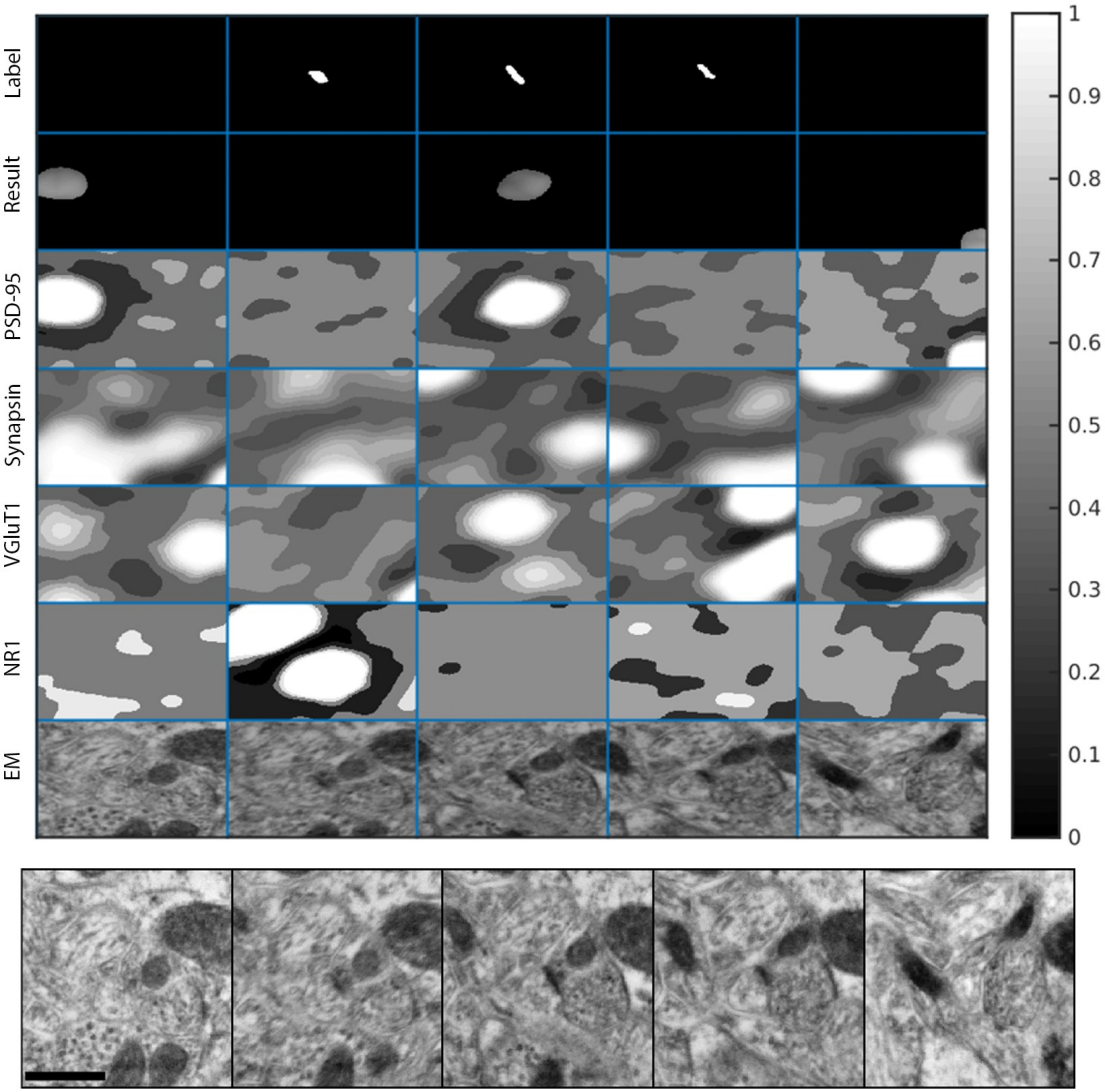
False positive synaptogram. Synaptogram showing a ‘false positive.’ Presynaptic and postsynaptic proteins are visible and experts would often label this a synapse presented with IF data alone, but no synapse was identified in the corresponding EM section. The algorithm makes the same mistake as a human expert. Each ‘block’ is 1.069*μ* m ×1.011*μ* m. As before, the bottom panel shows enlarged, consecutive slices of the EM data, which was used to manually annotate the synapse. The scale bar on the lower left side is 500 nm.

**Fig 13:**
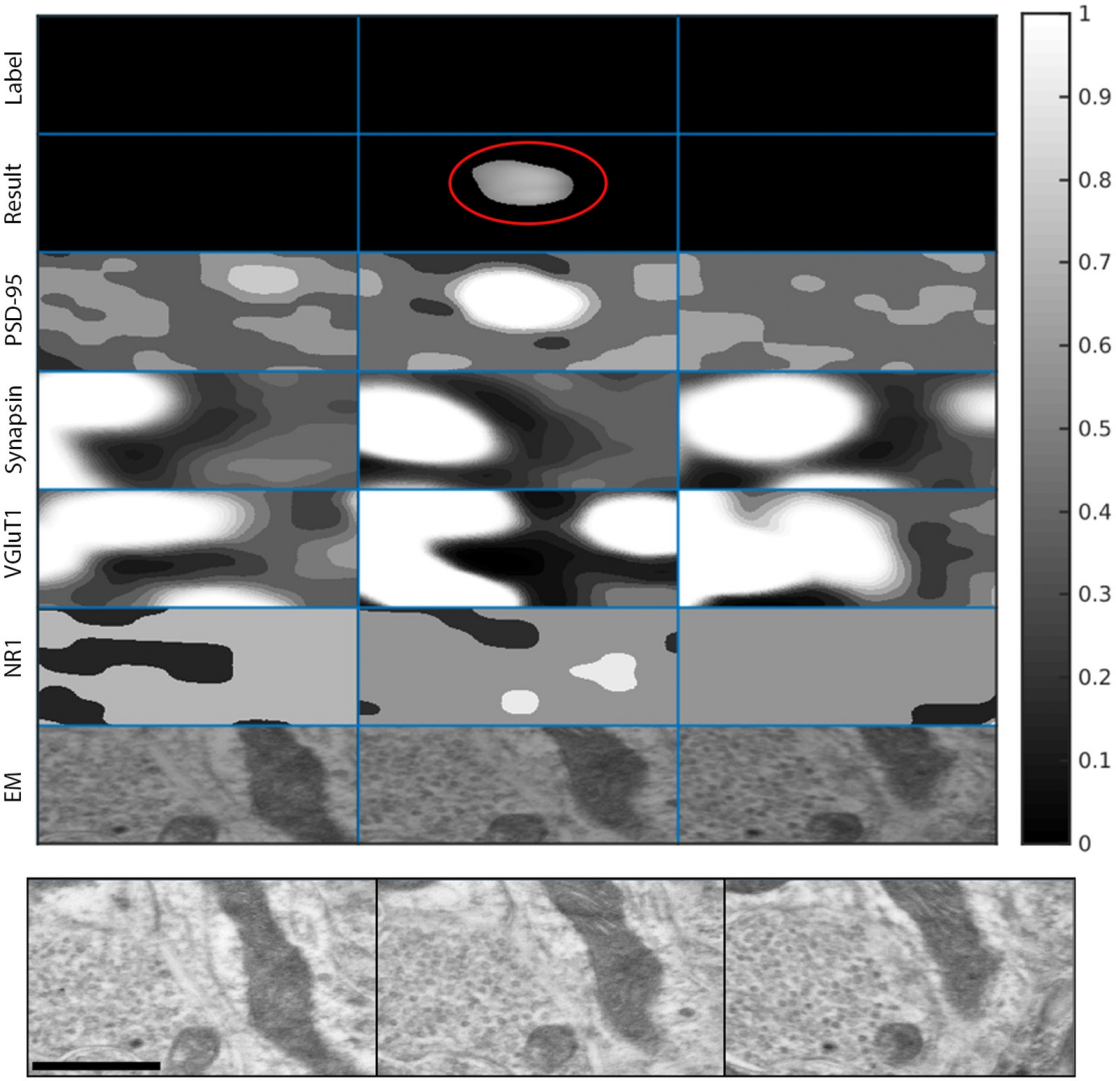
False negative synaptogram. Synaptogram showing a ‘false negative.’ While the corresponding EM sections shows a synapse, there is insuficient synaptic IF signal available to justify the presence of a synapse using solely IF data. Again, the algorithm makes the same mistake a human expert would make when working only with the IF data. Each ‘block’ is 1.086*μ* m ×1.130*μ* m. As before, the bottom panel shows enlarged, consecutive slices of the EM data, which was used to manually annotate the synapse. The scale bar on the lower left side is 500 nm.

Fig 11 shows an example of a true positive detection of excitatory synapses in the KDM-SYN-120905 dataset. The figure shows an example of a ‘synaptogram’, where each row (third to sixth rows) shows a different channel of immunofluorescent signal and each column is a 2D slice. The first row marked as *Label* shows the manual annotation of the synaptic cleft, i.e., the ground-truth, and the second row, marked as *Result*, corresponds to the output of the proposed synapse detection algorithm. Rows 3-6 are corresponding sections of each channel’s foreground probability map (the output of Step 2). The seventh row, marked as *EM*, shows the corresponding EM data. The panel below the synaptogram shows enlarged, consecutive slices of the EM data, which was used to manually annotate the synapse. Fig 12 shows an example of a false positive which cannot be differentiated from a real detection by an expert without the assistance of EM data (not available for our algorithm). Fig 13 shows a similar situation for a false negative detection.

### Evaluation on array tomography

The proposed method was evaluated on the array tomography dataset published in [19], which contains a portion of the mouse barrel cortex extending from Layer 3 to Layer 5. Unlike the conjugate array tomography dataset, there is no associated EM imagery. This larger series of datasets includes 11 volumes representing a total of 2,306,233 *μm*^3^ of cortical volume. Since no gold standard is available for these data, the proposed method was evaluated by verifying known properties of the dataset: there is an approximately ten-to-one ratio of excitatory to inhibitory synapses [24], and there are more inhibitory synapses in Layer 4 than Layer 5 in the mouse barrel cortex [25] [26] [27].

For this dataset, the query parameters were adjusted to reflect the different synaptic markers used. Tables 7 and 8 list the query parameters used for detecting both inhibitory and excitatory synapses, similar to those in [7] [8] [19].

**Table 7:** Excitatory synapse detection queries for the AT data.

Query	Presynaptic		Postsynaptic	
	Antibody	Puncta Size (x,y,z) *μm*	Antibody	Puncta Size (x,y,z) *μ* m
Query 1	synapsin	0.2 x 0.2 x 0.14	PSD-95	0.2 x 0.2 x 0.14
Query 2	synapsin VGluT1	0.2 x 0.2 x 0.14 0.2 x 0.2 x 0.07	PSD-95	0.2 x 0.2 x 0.07
Query 3	synapsin VGluT2	0.2 x 0.2 x 0.14 0.2 x 0.2 x 0.07	PSD-95	0.2 x 0.2 x 0.07
Query 4	synapsin GluR1	0.2 x 0.2 x 0.14 0.2 x 0.2 x 0.14	PSD-95	0.2 x 0.2 x 0.07
Query 5	synapsin GluR2	0.2 x 0.2 x 0.14 0.2 x 0.2 x 0.07	PSD-95	0.2 x 0.2 x 0.07
Query 6	synapsin	0.2 x 0.2 x 0.07	PSD-95 NR2B	0.2 x 0.2 x 0.07 0.2 x 0.2 x 0.07

**Table 8:** Inhibitory synapse detection queries for the AT data.

Query	Presynaptic		Postsynaptic	
	Antibody	Puncta Size (x,y,z) *μm*	Antibody	Puncta Size (x,y,z) *μ* m
Query 1	synapsin	0.2 x 0.2 x 0.07	gephyrin GABAAR	0.2 x 0.2 x 0.07 0.2 x 0.2 x 0.07
Query 2	synapsin vGAT	0.2 x 0.2 x 0.07 0.2 x 0.2 x 0.07	gephyrin	0.2 x 0.2 x 0.07
Query 3	synapsin GAD	0.2 x 0.2 x 0.07 0.2 x 0.2 x 0.07	gephyrin	0.2 x 0.2 x 0.07

#### Thresholding the probability map

**Fig 14:**
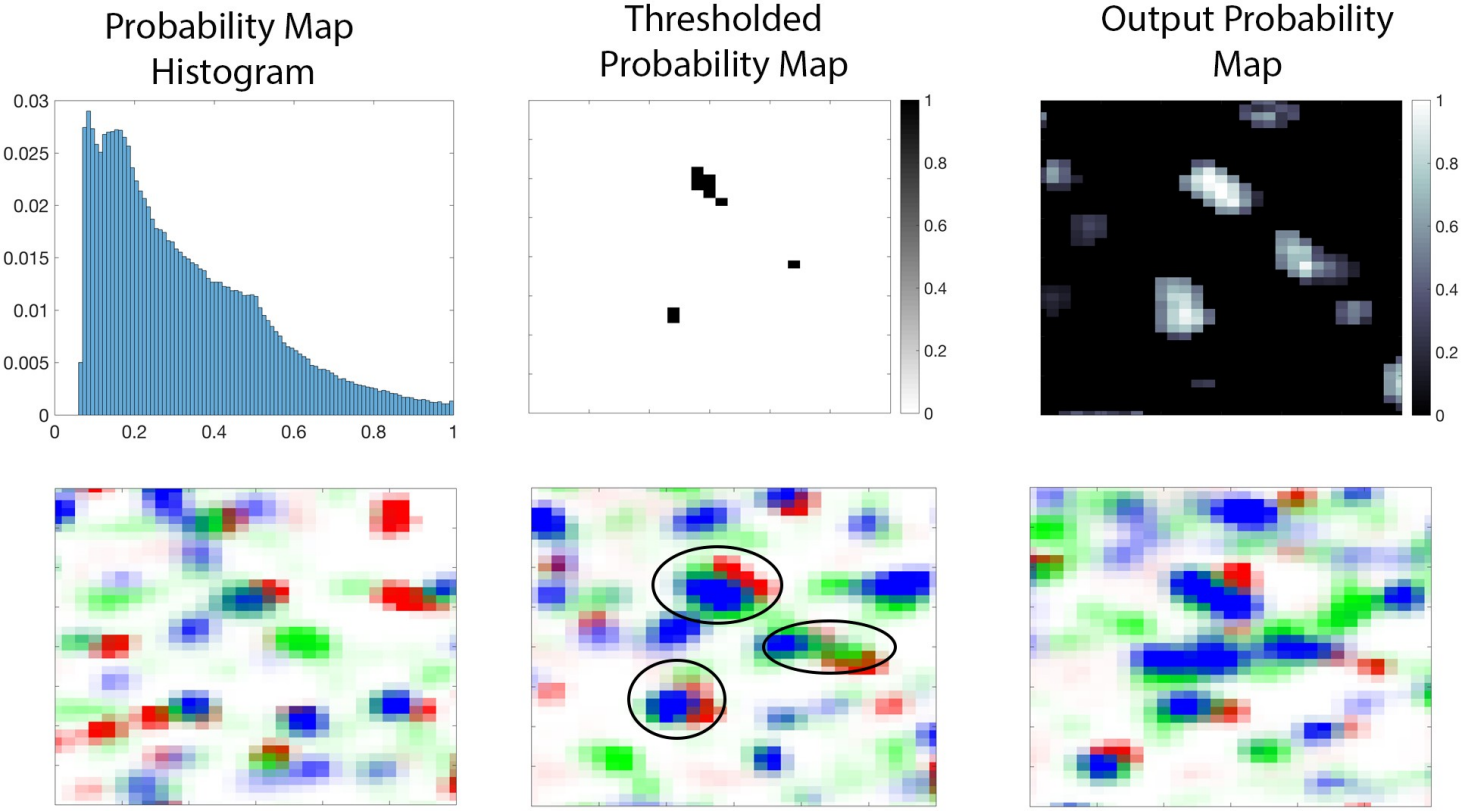
Synapse detection results. **Top Left:** Histogram of a slice of the probability map. **Top Middle:** Thresholded probability map, see text for details on the threshold selection. **Top right:** Probability map of one slice of a 13 *μ* m ×17 *μ* m region of the AT data on [19]. **Bottom Row:**. A series of pseudo-color images indicating the presence of the receptors PSD-95 (red), VGluT1 (blue), and synapsin (green) across three consecutive slices. Centroids of detected synapses containing all three receptors (query) are circled in black.

**Fig 15:**
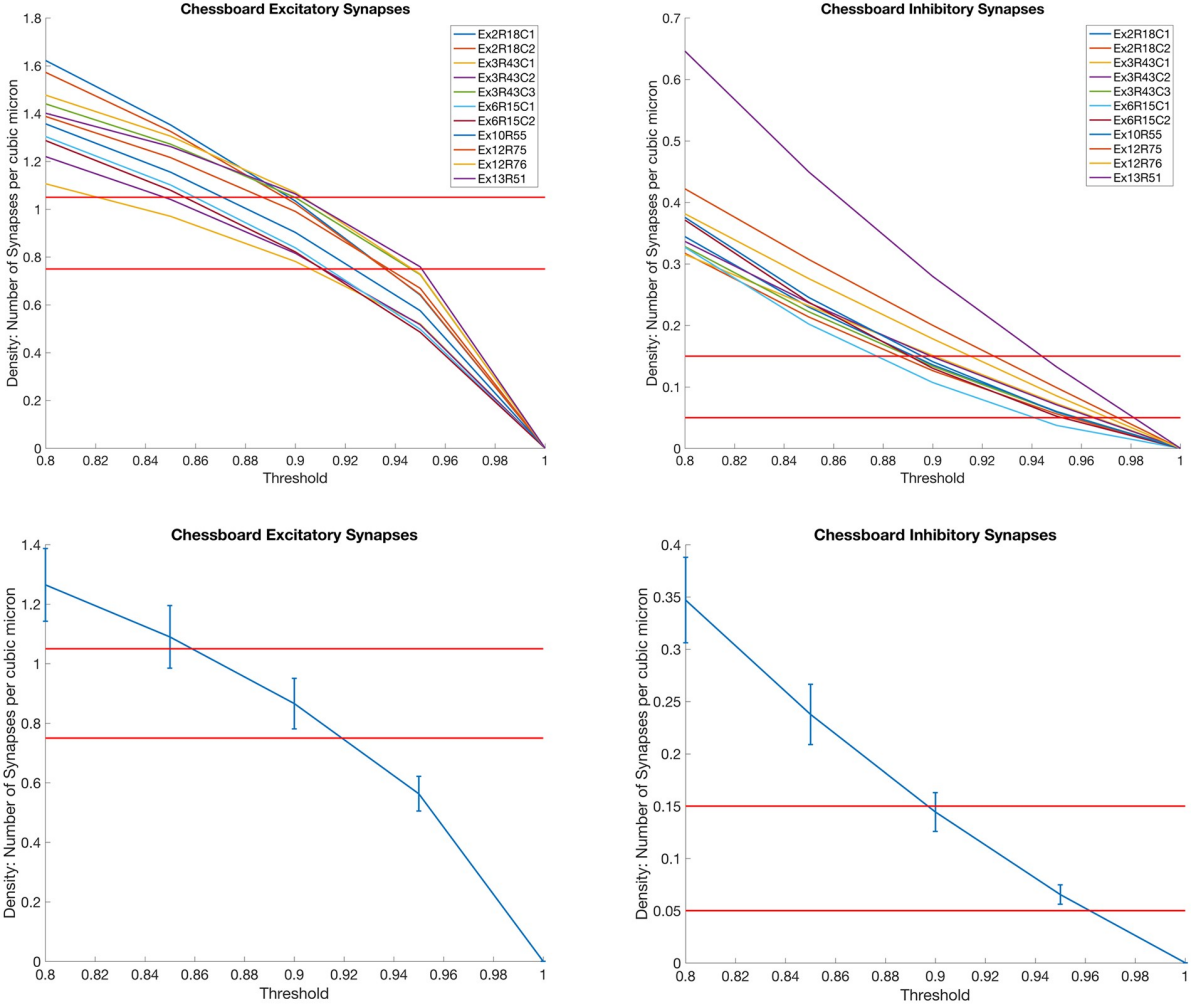
Thresholds vs Density. Plots showing the variation of putative synapse density across different thresholds. In this first row, each curve represents a dataset in [19] and the red lines show the expected synaptic density. For excitatory synapses, the expected density is 0.9 synapses *μ* m^3^ ± 0.15*μ*m^3^. For inhibitory synapses, the expected density is 0.1 synapses *μ* m^3^ ± 0.05*μ*m^3^ [28] [29]. The first row shows the relationship between density and the threshold for each dataset, while the second row shows the average density of all the datasets as a function of the threshold. The error bars represent the standard error.

Once the probability maps were computed (Fig 14), they were thresholded for evaluation purposes only. Thresholds for each dataset were determined by examining the synaptic density values across various thresholds, as shown in Fig 15. As the figure shows, the appropriate thresholds for each dataset exist in a narrow band, consistent with the results in the cAT dataset. Thresholding at the optimal value shown in Fig 15 for each dataset, as set by plots in Fig 15, amounted to 2,326,692 excitatory synapses and 252,833 inhibitory synapses. This amounts to approximately 1.12 synapses per cubic micrometer and an overall ratio of 9.2 excitatory to inhibitory synapses, which is consistent with results in the literature [30] [29].

**Fig 16:**
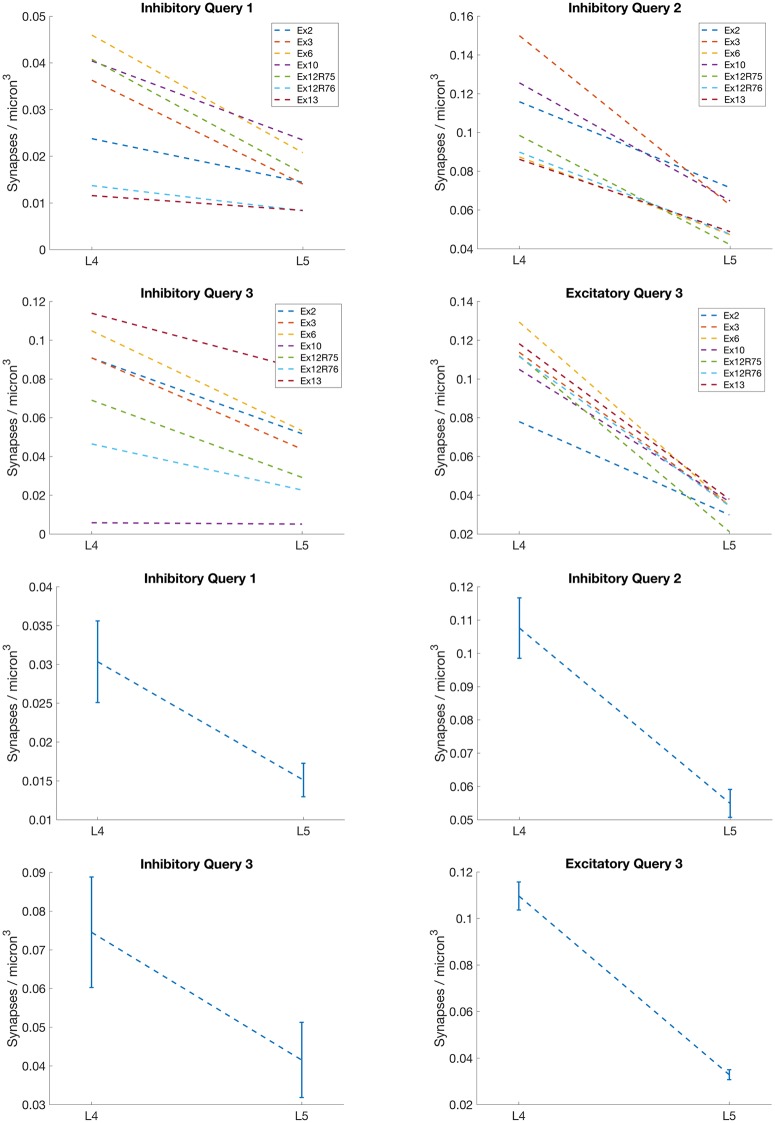
Mouse barrel cortex layer density differences. Plots showing the difference in synaptic density between Layer IV and Layer V for specific queries. The first two rows show the difference in density for each dataset. The last two rows show the average difference in density across all the datasets. The error bars represent the standard error. Each dataset was thresholded using the ‘optimal’ threshold value as determined from Fig 15.

Previous quantitative electron microscopy indicates that the synapse density should be higher in Layer IV than in Layer V [31], consistent with the results from our algorithm, as shown in the graphs in Fig 16. For all three inhibitory synaptic queries, there is a synapse density difference of more than 50% between Layer IV and Layer V. There is also a greater than 50% synaptic density difference between Layer IV and Layer V for excitatory synapses containing VGluT2, as supported by [7] [32] [33] [34]. These results further support the validity of the proposed method by confirming known biological properties of a large dataset.

The threshold of the estimated probability can be set to optimize a specific desired property (density in this case), thereby becoming an additional ‘query.’ The threshold can actually add flexibility, since different thresholds might lead to selective detection of different types of synapses. This possibility will be studied when new data becomes available, now that the unsupervised algorithm here introduced can be applied to such data (previous algorithms were basically limited to making binary decisions for detecting synapses they have been trained to detect). This means that the only non-straightforwardly physical parameter of the proposed algorithm (virtually all image processing algorithms have critical parameters) can add flexibility to the technique. Finally, the threshold can be ignored if we work directly with the probability map Eq (8), e.g., to compute ‘fuzzy volumes.’ This unique aspect of the proposed algorithm output will also be the subject of study when running the algorithm on new AT data in the future.

Thresholding in [8] was done via manual inspection to get the average punctum size of 0.09 um^2^, a number found by [8] to be the most effective PSD-95 size for synapse detection. This value might change with new data protocols or different antibody markers, thereby requiring new supervised training. We replaced the thresholding step with a step which computes the foreground probability of each pixel. Approximately 2% of the IF data volume is foreground, the rest is background. Therefore, we model the probability of a pixel belonging to the background using a Gaussian to model the entire dataset as the background. The proposed system permits to change this threshold per other biological (or instrument or antibody) queries.

## Discussion

The proposed synapse detection method serves the potential future needs of both basic and clinical neuroscience. Methods for large-scale synapse detection could analyze image volumes large enough to contain complete neural arbors, and thus allow the discernment of the relationships between detected synapses and their presynaptic and postsynaptic parent neurons. An understanding of the statistics of synapse variation in any given synaptic network is certain to be critical to interpreting and modeling results of mechanistic physiological study. Moreover, advances in imaging methods for tracing complete axonal and dendritic arbors [35] are likely to allow network analyses at the level of individual neurons and their synaptic connections, which might be optimally detected and measured by probabilistic means like those introduced here. When combined with complete arbor measurements [36], emerging methods for in situ measurement of single-cell transcriptomes [37] should allow single-synapse measurements to be associated with specific presynaptic and postsynaptic parent neurons of known transcriptomic profiles. Such capacities are likely to enhance our understanding of the molecular origins of synapse diversity. On the clinical side, the analysis tool we introduce here is likely to advance our abilities to detect possible abnormalities of synapse population statistics that have long been hypothesized to underlie a wide variety of mental and neurological disorders [38] [39] [40] [41] [42] [43] [44]. Quantification of synapse populations, in human postmortem and biopsy specimens, [45], and in animal models of disease, has already provided important insights into disease etiologies [39] [45] [42]. More reliable measurements based on probabilistic tools like those introduced here seem likely to facilitate future efforts to better understand disease mechanisms and to develop the quantitative assays essential to the discovery of effective therapies [46].

This work introduces a model-based unsupervised synapse detection algorithm that incorporates fundamental biological knowledge of how synapses are identified in the immunofluorescence data. We created a series of probabilistic excitatory detectors for various subtypes of synapses, and included the 3D spatial relationships typical of synaptic structures. This novel approach provides a probabilistic-based detection algorithm yielding not only detection but detection with confidence values. The implementation of synapse detection as a probability map (i.e., probability of each pixel belonging to a synapse), as opposed to a binary detection / no-detection result may provide a powerful tool to assist experts throughout the exploratory process to gain new insights from the immunofluorescence data, including potentially discovering new subtypes of synapses. The influence of different biological and AT components on the actual probability values, from the noise of the system to the expression level of the proteins and the subclass of the synapses, an important new topic of investigation, will become possible when the proposed algorithm is applied to large new datasets, currently being generated. Creating conjugate Array Tomography datasets require specialized equipment, including the use of a Field Emission Scanning Electron Microscope (FESEM) to provide ground truth validation of synapse results. The computational work presented in this paper, together with the publicly available code and data, is a step in the direction of making this kind of analysis robust enough to no longer require expensive FESEM validation.

The algorithm is computationally very simple and the only parameters are the user’s definition of a synapse subtype, rendering it ready for massive datasets. The results were validated with the best available cAT and AT data, producing state-of-the-art results without the need for supervised training. As demonstrated here, the proposed framework can be exploited for the explicit detection of synapses or their properties, the latter being critical for the discovery of new subtypes as well as the patterns of distributions of known subtypes. These, and the potential consequences of the approach here proposed to other modalities, are the subjects of our current efforts.

## References

[1] Broadhead MJ, Horrocks MH, Zhu F, Muresan L, Benavides-Piccione R, DeFelipe J, et al PSD95 nanoclusters are postsynaptic building blocks in hippocampus circuits. Scientific reports. 2016;6 10.1038/srep24626 27109929 PMC4842999

[2] Emes RD, Grant SG. Evolution of synapse complexity and diversity. Annual review of neuroscience. 2012;35:111–131. 10.1146/annurev-neuro-062111-150433 22715880

[3] O’rourke NA, Weiler NC, Micheva KD, Smith SJ. Deep molecular diversity of mammalian synapses: why it matters and how to measure it. Nature Reviews Neuroscience. 2012;13(6):365–379. 10.1038/nrn3170 22573027 PMC3670986

[4] Zampini V, Liu JK, Diana MA, Maldonado PP, Brunel N, Dieudonné S. Mechanisms and functional roles of glutamatergic synapse diversity in a cerebellar circuit. eLife. 2016;5:e15872 10.7554/eLife.15872 27642013 PMC5074806

[5] Roncal WG, Pekala M, Kaynig-Fittkau V, Kleissas DM, Vogelstein JT, Pfister H, et al. VESICLE: Volumetric evaluation of synaptic interfaces using computer vision at large scale. arXiv preprint arXiv:14033724. 2014;.

[6] Bloss EB, Cembrowski MS, Karsh B, Colonell J, Fetter RD, Spruston N. Structured dendritic inhibition supports branch-selective integration in CA1 pyramidal cells. Neuron. 2016;89(5):1016–1030. 10.1016/j.neuron.2016.01.029 26898780

[7] Busse B, Smith S. Automated analysis of a diverse synapse population. PLoS Comput Biol. 2013;9(3):e1002976 10.1371/journal.pcbi.1002976 23555213 PMC3610606

[8] Collman F, Buchanan J, Phend KD, Micheva KD, Weinberg RJ, Smith SJ. Mapping synapses by conjugate light-electron array tomography. Journal of Neuroscience. 2015;35(14):5792–5807. 10.1523/JNEUROSCI.4274-14.2015 25855189 PMC4388933

[9] Wang G, Smith SJ. Sub-diffraction limit localization of proteins in volumetric space using Bayesian restoration of fluorescence images from ultrathin specimens. PLoS Comput Biol. 2012;8(8):e1002671 10.1371/journal.pcbi.1002671 22956902 PMC3431294

[10] Wang GX, Smith SJ, Mourrain P. Sub-synaptic, multiplexed analysis of proteins reveals Fragile X related protein 2 is mislocalized in Fmr1 KO synapses. eLife. 2016;5:e20560 10.7554/eLife.20560 27770568 PMC5098911

[11] Burette A, Collman F, Micheva KD, Smith SJ, Weinberg RJ. Knowing a synapse when you see one. Frontiers in neuroanatomy. 2015;9 10.3389/fnana.2015.00100 26283929 PMC4517447

[12] Merchan-Perez A, Rodriguez JR, AlonsoNanclares L, Schertel A, DeFelipe J. Counting synapses using FIB/SEM microscopy: a true revolution for ultrastructural volume reconstruction. Frontiers in neuroanatomy. 2009;3:18 10.3389/neuro.05.018.2009 19949485 PMC2784681

[13] Neila PM, Baumela L, González-Soriano J, Rodríguez JR, DeFelipe J, Merchán-Pérez Á. A fast method for the segmentation of synaptic junctions and mitochondria in serial electron microscopic images of the brain. Neuroinformatics. 2016;14(2):235–250. 10.1007/s12021-015-9288-z 26780198 PMC4823374

[14] Lichtman JW, Denk W. The big and the small: challenges of imaging the brain’s circuits. Science. 2011;334(6056):618–623. 10.1126/science.1209168 22053041

[15] Rah JC, Feng L, Druckmann S, Lee H, Kim J. From a meso-to micro-scale connectome: array tomography and mGRASP. Frontiers in neuroanatomy. 2015;9:78 10.3389/fnana.2015.00078 26089781 PMC4454886

[16] Swanson LW, Lichtman JW. From Cajal to connectome and beyond. Annual Review of Neuroscience. 2016;39:197–216. 10.1146/annurev-neuro-071714-033954 27442070

[17] da Costa NM, Hepp K, Martin KA. A systematic random sampling scheme optimized to detect the proportion of rare synapses in the neuropil. Journal of neuroscience methods. 2009;180(1):77–81. 10.1016/j.jneumeth.2009.03.001 19427532

[18] Harris KM, Weinberg RJ. Ultrastructure of synapses in the mammalian brain. Cold Spring Harbor perspectives in biology. 2012;4(5):a005587 10.1101/cshperspect.a005587 22357909 PMC3331701

[19] Weiler NC, Collman F, Vogelstein JT, Burns R, Smith SJ. Synaptic molecular imaging in spared and deprived columns of mouse barrel cortex with array tomography. Scientific data. 2014;1 10.1038/sdata.2014.46 25977797 PMC4411012

[20] Micheva KD, Smith SJ. Array tomography: a new tool for imaging the molecular architecture and ultrastructure of neural circuits. Neuron. 2007;55(1):25–36. 10.1016/j.neuron.2007.06.014 17610815 PMC2080672

[21] Dosemeci A, Makusky AJ, Jankowska-Stephens E, Yang X, Slotta DJ, Markey SP. Composition of the synaptic PSD-95 complex. Molecular & Cellular Proteomics. 2007;6(10):1749–1760. 10.1074/mcp.M700040-MCP200 17623647 PMC2096750

[22] Korogod N, Petersen CC, Knott GW. Ultrastructural analysis of adult mouse neocortex comparing aldehyde perfusion with cryo fixation. Elife. 2015;4:e05793 10.7554/eLife.05793 PMC4530226 26259873

[23] Brown LD, Cai TT, DasGupta A. Interval estimation for a binomial proportion. Statistical science. 2001; p. 101–117.

[24] Knott GW, Quairiaux C, Genoud C, Welker E. Formation of dendritic spines with GABAergic synapses induced by whisker stimulation in adult mice. Neuron. 2002;34(2):265–273. 10.1016/S0896-6273(02)00663-3 11970868

[25] De Felipe J, Marco P, Fairen A, Jones E. Inhibitory synaptogenesis in mouse somatosensory cortex. Cerebral Cortex. 1997;7(7):619–634. 10.1093/cercor/7.7.619 9373018

[26] Knott GW, Holtmaat A, Wilbrecht L, Welker E, Svoboda K. Spine growth precedes synapse formation in the adult neocortex in vivo. Nature neuroscience. 2006;9(9):1117–1124. 10.1038/nn1747 16892056

[27] Micheva K, Beaulieu C. Development and plasticity of the inhibitory neocortical circuitry with an emphasis on the rodent barrel field cortex: a review. Canadian journal of physiology and pharmacology. 1997;75(5):470–478. 10.1139/y97-032 9250380

[28] Calverley R, Jones D. A serial-section study of perforated synapses in rat neocortex. Cell and tissue research. 1987;247(3):565–572. 10.1007/BF00215750 3568103

[29] Schüz A, Palm G. Density of neurons and synapses in the cerebral cortex of the mouse. Journal of Comparative Neurology. 1989;286(4):442–455. 10.1002/cne.902860404 2778101

[30] Beaulieu C, Campistron G, Crevier C. Quantitative aspects of the GABA circuitry in the primary visual cortex of the adult rat. Journal of Comparative Neurology. 1994;339(4):559–572. 10.1002/cne.903390407 8144746

[31] Micheva KD, Beaulieu C. An anatomical substrate for experience-dependent plasticity of the rat barrel field cortex. Proceedings of the National Academy of Sciences. 1995;92(25):11834–11838. 10.1073/pnas.92.25.11834 PMC40497 8524859

[32] Graziano A, Liu XB, Murray KD, Jones EG. Vesicular glutamate transporters define two sets of glutamatergic afferents to the somatosensory thalamus and two thalamocortical projections in the mouse. Journal of comparative neurology. 2008;507(2):1258–1276. 10.1002/cne.21592 18181146

[33] Nakamura K, Watakabe A, Hioki H, Fujiyama F, Tanaka Y, Yamamori T, et al Transiently increased colocalization of vesicular glutamate transporters 1 and 2 at single axon terminals during postnatal development of mouse neocortex: a quantitative analysis with correlation coefficient. European Journal of Neuroscience. 2007;26(11):3054–3067. 10.1111/j.1460-9568.2007.05868.x 18028110

[34] Rah JC, Bas E, Colonell J, Mishchenko Y, Karsh B, Fetter RD, et al Thalamocortical input onto layer 5 pyramidal neurons measured using quantitative large-scale array tomography. Frontiers in neural circuits. 2013;7:177 10.3389/fncir.2013.00177 24273494 PMC3824245

[35] Bosch C, Martínez A, Masachs N, Teixeira CM, Fernaud I, Ulloa F, et al FIB/SEM technology and high-throughput 3D reconstruction of dendritic spines and synapses in GFP-labeled adult-generated neurons. Frontiers in neuroanatomy. 2015;9:60 10.3389/fnana.2015.00060 26052271 PMC4440362

[36] Chen F, Wassie AT, Cote AJ, Sinha A, Alon S, Asano S, et al Nanoscale imaging of RNA with expansion microscopy. Nature Methods. 2016;13(8):679–684. 10.1038/nmeth.3899 27376770 PMC4965288

[37] Moffitt JR, Hao J, Bambah-Mukku D, Lu T, Dulac C, Zhuang X. High-performance multiplexed fluorescence in situ hybridization in culture and tissue with matrix imprinting and clearing. Proceedings of the National Academy of Sciences. 2016; p. 201617699. 10.1073/pnas.1617699113 PMC5167177 27911841

[38] Davies C, Mann D, Sumpter P, Yates P. A quantitative morphometric analysis of the neuronal and synaptic content of the frontal and temporal cortex in patients with Alzheimer’s disease. Journal of the neurological sciences. 1987;78(2):151–164. 10.1016/0022-510X(87)90057-8 3572454

[39] Grant SG. Synaptopathies: diseases of the synaptome. Current opinion in neurobiology. 2012;22(3):522–529. 10.1016/j.conb.2012.02.002 22409856

[40] Guilmatre A, Huguet G, Delorme R, Bourgeron T. The emerging role of SHANK genes in neuropsychiatric disorders. Developmental neurobiology. 2014;74(2):113–122. 10.1002/dneu.22128 24124131

[41] Kobel M Le Prell CG Liu J Hawks JW Bao J. Noise-induced cochlear synaptopathy: Past findings and future studies. Hearing Research. 2016;. 10.1016/j.heares.2016.12.008 28007526

[42] Sheng M, Sabatini BL, Südhof TC. Synapses and Alzheimer’s disease. Cold Spring Harbor perspectives in biology. 2012;4(5):a005777 10.1101/cshperspect.a005777 22491782 PMC3331702

[43] Terry RD, Masliah E, Salmon DP, Butters N, DeTeresa R, Hill R, et al Physical basis of cognitive alterations in Alzheimer’s disease: synapse loss is the major correlate of cognitive impairment. Annals of neurology. 1991;30(4):572–580. 10.1002/ana.410300410 1789684

[44] Verpelli C, Sala C. Molecular and synaptic defects in intellectual disability syndromes. Current opinion in neurobiology. 2012;22(3):530–536. 10.1016/j.conb.2011.09.007 22000839

[45] Henstridge CM, Pickett E, Spires-Jones TL. Synaptic pathology: a shared mechanism in neurological disease. Ageing research reviews. 2016;28:72–84. 10.1016/j.arr.2016.04.005 27108053

[46] Fitzsimmons J, Kubicki M, Shenton ME. Review of functional and anatomical brain connectivity findings in schizophrenia. Current opinion in psychiatry. 2013;26(2):172–187. 10.1097/YCO.0b013e32835d9e6a 23324948

